# The composition of the aphid fauna (Insecta, Hemiptera) of the Royal Botanic Gardens, Kew

**DOI:** 10.1038/s41598-019-46441-z

**Published:** 2019-07-10

**Authors:** Karina Wieczorek, Tim K. Fulcher, Dominik Chłond

**Affiliations:** 1 Department of Zoology, Faculty of Biology and Environmental Protection, University of Silesia, Bankowa 9, 40-007 Katowice, Poland; 2 Royal Botanic Gardens, Kew, London UK

**Keywords:** Biodiversity, Entomology

## Abstract

At least a dozen species of aphids (Insecta, Hemiptera) of non-native origin have expanded their range in Europe, however the importance of botanic gardens in this phenomenon has not been studied previously in detail. As a case study, investigations on the species composition and host range of Aphidomorpha in the Royal Botanic Gardens, Kew, London, United Kingdom, were conducted over a period of twelve days, in June 2017. The inventory study was carried out in the collection of living plants, both in the gardens and the glasshouses and nurseries. In total, 94 taxa of Aphidomorpha are identified (one phylloxerid, one adelgid and 92 species of aphids). 20 species are regarded as alien to the European aphid fauna and among them nine are believed to be the first published records for Kew. 20 species are regarded as serious pests, capable of virus transmission. The list of host plants includes 155 taxa from 89 genera and 49 families. *Ericolophium holsti* (Takahashi), species of Asiatic origin associated with *Rhododendron* spp., was found for the first time in the field in the UK. Changes in the species composition of the aphid fauna in reference to the Eastop’s studies in 1960s were discussed.

## Introduction

Aphids, and closely related phylloxerids and adelgids (Insecta: Hemiptera: Aphidomorpha), are one of the most important groups of pests on cultivated and ornamental plants in the temperate regions. Possible effects include weakening and distortion of host plants, decreased growth rates, secretion of large amounts of honeydew and the transfer of plant viruses^1,2^. The fight against these insects is difficult due to their biology – holocyclic or, in some species, anholocyclic (i.e. without sexual phase) mode of reproduction and extremely high female fecundity (e.g. the peach-potato aphid *Myzus persicae* (Sulzer)^3^ or the soybean aphid *Aphis glycines* Matsumura^4^). Another important feature is the host alternation: the presence of various generations in one season, including winged morphs responsible for dispersal and locating secondary (or new) hosts^2^. Intraspecific variation (e.g. in expression of sexuality in the bird cherry-oat aphid *Rhopalosiphum padi* L.^5^), the way in which foraging affects the physiology of the plants infested (including the influence of virus-induced changes^6^) and the observed lack of susceptibility to some insecticides^7^, are additional factors, which have enabled aphids to exploit their food-plants. The species of non-native origin play a special role, especially in new areas, and under favourable conditions can become invasive^8,9^ or can attack native^10^ or endemic plants^11^. As many as 102 alien aphid species have been reported in Europe^12^. However, this number is continuously changing due to the increasing globalization of trade in plants and plant material, together with climate change^13,14^. Consequently, it leads to an increase in the introduction and spread of new and damaging plant pests and pathogens, causing serious losses in plant production^15–19^. On the other hand, the distribution of these insects is limited by the presence of the host plants, i.e. the alien aphids are absent where the host plant does not occur. Some alien aphids were introduced with the exotic host^20,21^, thus, these (at least in some cases), are restricted to artificial habitats such as botanic gardens, greenhouses, parks and gardens in city areas. Among them, botanic gardens are classified as the oldest form of urban greenery, covering all aspects of plant conservation policy, practice and education and characterized by high plant diversity^22^. At the same time, botanic gardens are a small but significant part of the invasive plant problem^23–26^. However, their role in the spread of an organism as closely associated with the host plant as Aphidomorha, has not been sufficiently studied.

Created in 1759, the Royal Botanic Gardens, Kew (Kew), across its 132 hectares, grows one of the largest and most diverse living plant collections in the world. This is London’s largest UNESCO World Heritage Site, designated in 2003, with more than 100,000 living plants. These represent numerous and diverse plant families with extensive collections of trees, herbaceous, alpine and economic plants from most parts of the world, located in distinctive areas and glasshouses, and as such is an excellent target for collecting Aphidomorpha. The first list of aphids, collected by Laing in Kew, was published in 1920^27^. Later, in 1962 and 1965, further contributions to the aphid fauna of Kew were published by Eastop^28,29^. The first list brings the total number of aphids known from Kew to 91 taxa, the second one comprised 77 taxa. In total, over four years of collecting (1958, 1960, 1961 and 1962), 142 taxa of aphids were listed. Some of them were marked as introduced or even as a first record for Europe. With a few minor exceptions^30–32^, further intensive studies on the aphid fauna of Kew have not been carried out.

The aim of the paper is to ascertain the number of Aphidomorpha species infesting plants in Kew and see how the aphid fauna has changed since Eastop’s research 60 years ago. Moreover, it will allow identification of non-native species and whether the number of introductions of aliens has changed. It will also determine the number of economically important species of aphids.

## Results

### The composition of the Aphidomorpha fauna of the Royal Botanic Gardens, Kew

A total of 221 aphid samples were collected during the twelve days in Kew. In total, 94 taxa of Aphidomorpha were identified. Adelgidae and Phylloxeridae were represented by a single species each, whereas there were 92 taxa from Aphididae (species and subspecies) belonging to nine subfamilies: Eriosomatinae, Anoeciinae, Mindarinae, Drepanosiphinae, Phyllaphidinae, Calaphidinae, Chaitophorinae, Aphidinae and Lachninae. Aphidinae, the most numerous subfamily, was represented by 20 genera and 44 species. The richest genus represented in the collection was *Aphis* Linnaeus (11 species). The subfamily Calaphidinae (21 species) was most frequently represented by species belonging to the tribe Panaphidini (14 species). The fewest species in Aphididae were from the subfamilies Anoeciinae, Mindarinae, Drepanosiphinae and Phyllaphidinae.

**Figure 1:**
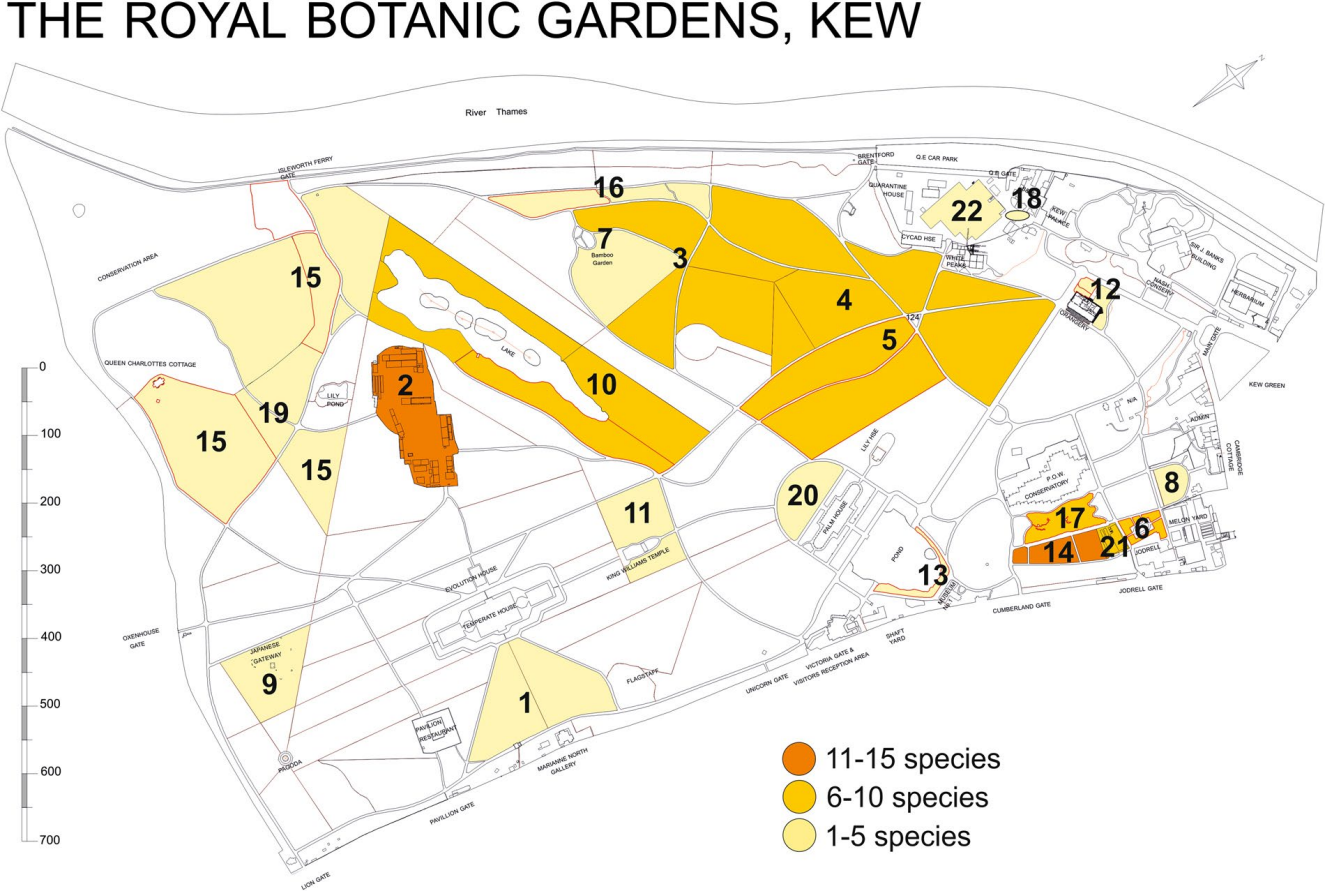
Collecting area and the number of collected species. The abbreviations denote as follow: (1) AColl. – *Acer* spp. collection; (2) ARBN – Arboretum Nursery; (3) BColl. – *Betula* spp. collection; (4) PColl. – *Populus* spp. collection; (5) QColl. – *Quercus* spp. collection; (6) AG – Aquatic Garden; (7) BG – Bamboo Garden; (8) DG – Duke’s Garden; (9) JG – Japanese Garden; (10) L – Lake; (11) MG – Mediterranean Garden; (12) NO – near Orangery; (13) P – Pond; (14) PFB – Plant Family Beds; (15) Pi – Pinetum; (16) RD – Rhododendron Dell; (17) RG – Rock Garden; (18) RK – Royals Kitchen; (19) ReG – Redwood Grove; (20) RoG – Rose Garden; (21) SVP – Student Vegetables Plot; (22) TRON – Tropical Nursery.

In only seven locations surveyed (Aquatic Garden, Duke’s Garden, Lake, Mediterranean Garden, Pond, Rhododendron Dell, Redwood Grove) the number of species was the same as the number of samples collected. In other locations, the number of samples was slightly higher than the number of species, the highest in the *Populus* collection (5 species and 14 samples). The two richest locations for aphid species and samples were the Arboretum Nursery (15 species and 17 samples) and the Plant Family Beds (12 species and 16 samples). In other locations, from 1 to 10 species (Fig. 1) and 1 to 14 samples were found. In these locations, all samples were collected in outdoor conditions, except for a few samples collected in a greenhouses in Arboretum Nursery or Tropical Nursery. In contrast, in indoor conditions in the Palm House, Water Lily House, Princess of Wales Conservatory or Davies Alpine House no samples were collected.

### Alien aphid species

20 species were regarded as alien to the European aphid fauna and among them nine are believed to be the first published records for Kew: *Aphis* (*Aphis*) *gossypii* Glover, 1877, *A*. (*A*.) *spiraecola* Patch, 1914, *A*. (*Toxoptera*) *aurantii* Boyer de Fonscolombe, 1841, *Chaetosiphon* (*Pentatrichopus*) *fragaefolii* (Cockerell, 1901), *Ericolophium holsti* (Takahashi, 1935), *Illinoia* (*Illinoia*) *liriodendri* (Monell, 1879), *Illinoia* (*Masonaphis*) *lambersi* (MacGillivray, 1960), *Macrosiphum* (*Macrosiphum*) *albifrons* Essig, 1911, *Neotoxoptera formosana* (Takahashi, 1921).

### Pest species

20 species were regarded as serious pests, capable of virus transmission: *Aphis* (*Aphis*) *fabae* Scopoli, 1763, *A*. (*A*.) *gossypii*, *A*. (*A*.) *pomi* De Geer, 1773, *A*. (*A*.) *spiraecola*, *A*. (*Bursaphis*) *grossulariae* Kaltenbach, 1843, *A*. (*Toxoptera*) *aurantii*, *Hyalopterus pruni* (Geoffroy, 1762), *Rhopalosiphum nymphaeae* (Linnaeus, 1761), *Acyrthosiphon* (*Acyrthosiphon*) *pisum* Harris, 1776, *Brachycaudus* (*Prunaphis*) *cardui* (Linnaeus, 1758), *Cavariella aegopodii* (Scopoli, 1763), *Chaetosiphon* (*Pentatrichopus*) *fragaefolii*, *Cryptomyzus* (*Cryptomyzus*) *ribis* (Linnaeus, 1758), *Dysaphis* (*Dysaphis*) *tulipae* (Boyer de Fonscolombe, 1841), *Macrosiphum* (*Macrosiphum*) *euphorbiae* (Thomas, 1878), *M*. (*M*.) *rosae* (Linnaeus, 1758), *Megoura viciae* Buckton, 1876, *Myzus* (*Myzus*) *cerasi* (Fabricius, 1775), *M*. (*M*.) *ornatus* Laing, 1932, *M*. (*Nectarosiphon*) *persicae* (Sulzer, 1776).

Although the garden staff use mostly natural methods to control such serious pests (i.e. the biocontrols of different mixes of parasitoid wasps like *Aphidius colemani* Viereck, 1912, *A*. *ervi* Haliday, 1834, *A*. *matricariae* Haliday, 1834 *Aphelinus abdominalis* (Dalman, 1820), *Praon volucre* (Haliday, 1833), *Ephedrus cerasicola* Starý, 1962 and the predatory fly *Aphidoletes aphidimyza* (Rondani, 1847) or lace wing *Chrysoperla carnea* (Stephens, 1836) (P. Rees pers. comm.), without spreading aggressive insecticides, most infected host plants did not have visible damage. The exception were some plants in the Student Vegetables Plot e.g. *Prunus* sp., *Solanum lycopersicon*, *Solanum tuberosum* and in the Arboretum Nursery e.g. *Acer palmatum* ‘BiHoo’, *Ribes orientalis*, where feeding aphids promoted curled and distorted leaves as well as chlorosis or honeydew deposits. In the collection of living plants grown out in the Gardens feeding *Phylloxera glabra* (von Heyden, 1837) had caused necrotic spots on the leaves of *Quercus dentata* in the *Quercus* collection.

### Host-plants associations

The list of host plants includes 155 taxa from 89 genera and 49 families and is summarised in Table 1. The most frequently infested plant species belong to Fagaceae (20 species, 25 samples), Betulaceae and Sapindaceae (13 species and 15 samples each). The most frequently infested genera were *Quercus* (10 species, 21 samples) and *Acer* (6 species, 15 samples). The highest diversity of aphid species was observed on *Quercus* and consisted of 10 species. *Aphis* (*A*.) *fabae* and *Macrosiphum* (*M*.) *euphorbiae* were the most frequent aphid species found with the widest host range. Whereas *A*. (*A*.) *fabae* was found on colonized about 30 host plants, *M*. (*M*.) *euphorbiae* was only associated with ten hosts. The remaining species were associated with one to four host plants (Table 1).

**Table 1:** Host plant index and associated Aphidomorpha species collected in the Royal Botanic Gardens, Kew.

No.	Host plant taxon	Aphidomorpha taxon
1	*Abies pinsapo* Boiss.	*Mindarus abietinus*
2	*Acer campestre* L.	*Periphyllus hirticornis*, *Periphyllus lyropictus*
3	*Acer griseum* (Franch.) Pax	*Periphyllus acericola*
4	*Acer negundo* L.	*Periphyllus testudinaceus*
5	*Acer oblongum* Wall. ex DC.	*Drepanosiphum platanoidis*
6	*Acer oliverianum* Pax	*Periphyllus testudinaceus*
7	*Acer palmatum* Thunb.	*Periphyllus californiensis*
8	*Acer palmatum* Thunb. ‘Bi Hoo’	*Periphyllus californiensis*, *Periphyllus testudinaceus*
9	*Acer palmatum* Thunb. ‘Senkaki’	*Periphyllus testudinaceus*,
10	*Acer pseudoplatanus* L.	*Drepanosiphum platanoidis*
11	*Acer heldreichii* subsp. *trautvetteri* (Medw.) A.E. Murray	*Drepanosiphum platanoidis*, *Periphyllus acericola*
12	*Achillea millefolium* L. ‘Pink Grapefruit’	*Brachycaudus* (*Prunaphis*) *cardui*, *Macrosiphoniella* (*Macrosiphoniella*) *absinthii*
13	*Achillea* sp.	*Aphis* (*Aphis*) *fabae*, *Macrosiphoniella* (*Macrosiphoniella*) *millefolii*
14	*Acorus calamus* L.’Variegatus’	*Rhopalosiphum nymphaeae*
15	*Aesculus* × hybrida DC.	*Periphyllus testudinaceus*
16	*Aesculus turbinata* Blume	*Aphis* (*Aphis*) *fabae*
17	*Ageratina ligustrina* (DC.) R.M. King & H. Rob.	*Aphis* (*Aphis*) *fabae*
18	*Allium nutans* L.	*Neotoxoptera formosana*
19	*Alnus glutinosa* (L.) Gaertn.	*Pterocallis* (*Pterocallis*) *maculata*
20	*Alnus rubra* Bong.	*Pterocallis* (*Pterocallis*) *alni*
21	*Aquilegia vulgaris* L.	*Macrosiphum (Macrosiphum) euphorbiae*
22	*Arctium lappa* L.	*Aphis* (*Aphis*) *fabae*
23	*Artemisia absinthium* L.	*Macrosiphoniella* (*Macrosiphoniella*) *absinthii*
24	*Bambusa* sp.	*Takecallis arundinariae*
25	*Betula utilis subsp*. *albosinensis* (Burkill) Ashburner & McAll.	*Symydobius oblongus*
26	*Betula dauurica* Pall.	*Calaphis flava*
27	*Betula ermanii* Cham.	*Calaphis flava*
28	*Betula grossa* Siebold & Zucc.	*Callipterinella calliptera*
29	*Betula pubescens var*. *litwinowii* (Doluch.) Ashburner & McAll.	*Symydobius oblongus*
30	*Betula pendula* Roth	*Euceraphis betulae*
31	*Betula pendula* subsp. *mandshurica* (Regel) Ashburner & McAll.	*Clethrobius comes*
32	*Betula pendula* subsp. *szechuanica* (C.K. Schneid.) Ashburner & McAll.	*Euceraphis betulae*
33	*Betula utilis* D. Don	*Symydobius oblongus*
34	*Betula utilis* D. Don var. *prattii* Burkill	*Betulaphis quadrituberculata*, *Calaphis flava*, *Monaphis antennata*
35	*Bremeria landia* var. *holosericea* (Sm.) A.P. Davis & Razafim.	*Aphis* (*Aphis*) *spiraecola*
36	*Camellia japonica* L.	*Aphis* (*Toxoptera*) *aurantii*
37	*Carpinus cordata* Blume var. *chinensis* Franch.	*Myzocallis* (*Myzocallis*) *carpini*
38	*Castanea sativa* Mill.	*Myzocallis* (*Agrioaphis*) *castanicola*,
39	*Cedrus atlantica* (Endl.) Manetti ex Carrière	*Cinara* (*Cinara*) *cedri*
40	*Cedrus libani* A. Rich.	*Cinara* (*Cinara*) *cedri*
41	*Celastrus orbiculatus* Thunb.	*Aphis* (*Aphis*) *fabae*
42	*Cistus laurifolius* L.	*Aphis* (*Aphis*) *fabae*
43	*Clianthus puniceus* (G. Don) Sol. ex Lindl.	*Acyrthosiphon* (*Acyrthosiphon*) *malvae*
44	*Cornus mas* L.	*Macrosiphum* (*Macrosiphum*) *euphorbiae*
45	*Cornus* sp.	*Anoecia corni*
46	*Corylus avellana* L.	*Myzocallis* (*Myzocallis*) *coryli*, *Corylobium avellanae*
47	*Crataegus pentagyna* Waldst. & Kit. ex Willd.	*Aphis* (*Aphis*) *pomi*
48	*Crossandra pungens* Lindau	*Myzus* (*Nectarosiphon*) *persicae*
49	*Cynara cardunculus* L.	*Aphis* (*Aphis*) *fabae*
50	*Digitalis purpurea* L.	*Aphis* (*Aphis*) *fabae*
51	*Echium amoenum* Fisch. & C.A. Mey.	*Aphis* (*Aphis*) *fabae*
52	*Erythranthe naiandina* (J.M. Watson & C. Bohlen) G.L. Nesom	*Aphis* (*Aphis*) *fabae*
53	*Eschscholzia californica* Cham.	*Aphis* (*Aphis*) *fabae*
54	*Euphorbia characias* L.	*Macrosiphum (Macrosiphum) euphorbiellum*
55	*Fagus sylvatica* L.	*Phyllaphis fagi*
56	*Fagus sylvatica* ‘Tricolor’	*Phyllaphis fagi*
57	*Fatsia japonica* (Thunb) Decne. & Planch.	*Aphis* (*Aphis*) *fabae*
58	*Foeniculum vulgare* Mill.	*Cavariella aegopodii*
59	*Fragaria* × *ananassa* (Duchesne ex Weston) Duchesne ex Rozier	*Chaetosiphon* (*Pentatrichopus*) *fragaefolii*
60	*Hedera* sp.	*Aphis* (*Aphis*) *fabae*
61	*Hedlundia minima* (Ley) Sennikov & Kurtto	*Aphis* (*Aphis*) *pomi*
62	*Ilex* sp.	*Aphis* (*Aphis*) *ilicis*
63	*Iris pallida* Lam.	*Macrosiphum (Macrosiphum) euphorbiae*
64	*Iris* sp.	*Aphis* (*Aphis*) *newtoni*, *Rhopalosiphum nymphaeae*, *Dysaphis* (*Dysaphis*) *tulipae*, *Macrosiphum* (*Macrosiphum*) *euphorbiae*
65	*Juglans regia* L.	*Chromaphis juglandicola*, *Panaphis juglandis*
66	*Koelreuteria bipinnata* Franch.	*Aphis* (*Aphis*) *fabae*
67	*Lathyrus montanus* Bernh.	*Megoura viciae*
68	*Lathyrus* sp.	*Acyrthosiphon* (*Acyrthosiphon*) *pisum*
69	*Leptodermis pilosa* Diels	*Aphis* (*Aphis*) *gossypii*
70	*Leucanthemum* × *superbum* (Bergmans ex J.W.Ingram) D.H. Kent	*Aphis* (*Aphis*) *fabae*, *Brachycaudus (Prunaphis) cardui*
71	*Liriodendron tulipifera* L.	*Illinoia* (*Illinoia*) *liriodendri*
72	*Lonicera implexa* Aiton.	*Hyadaphis passerinii*
73	*Lupinus ehrenbergii* Schltdl. var. *ehrenbergii*	*Macrosiphum* (*Macrosiphum*) *albifrons*
74	*Lupinus* ‘My Castle’	*Macrosiphum* (*Macrosiphum*) *albifrons*
75	*Lupinus* ‘The Governor’	*Macrosiphum* (*Macrosiphum*) *albifrons*
76	*Lychnis coronaria* (L.) Desr.	*Brachycaudus* (*Acaudus*) *lychnidis*
77	*Malus domestica* (Sukow) Borkh.	*Dysaphis* (*Pomaphis*) *plantaginea*
78	*Malus tschonoskii* (Maxim.) C.K. Schneid.	*Aphis* (*Aphis*) *pomi*
79	*Matricaria chamomilla* L.	*Brachycaudus* (*Prunaphis*) *cardui*
80	*Monarda fistulosa* L. var *menthifolia* (Graham) Fernald	*Aphis* (*Aphis*) *fabae*
81	*Musa* sp.	*Aphis* (*Aphis*) *fabae*
82	*Nelumbo nucifera* Gaertn.	*Macrosiphum (Macrosiphum) euphorbiae*
83	*Oenothera magellanica* Phil.	*Aphis* (*Bursaphis*) *grossulariae*
84	*Oxylobium lineare* Benth.	*Aphis* (*Aphis*) *gossypii*
85	*Paulownia fargesii* Franch.	*Macrosiphum* (*Macrosiphum*) *euphorbiae*
86	*Phragmites australis* (Cav.) Trin. ex Steud.	*Hyalopterus pruni*
87	*Phyllostachys aurea* (André) Rivière & C. Rivière	*Takecallis arundinariae*
88	*Picea* sp.	*Adelges laricis*, *Cinara* (*Cinara*) *piceae*
89	*Pieris japonica* (Thunb.) D. Don ex G. Don	*Aphis* (*Aphis*) *fabae*
90	*Pinus nigra* J.F. Arnold	*Cinara* (*Cinara*) *pini*, *Cinara* (*Schizolachnus*) *pineti*
91	*Pinus patula* Schiede ex Schltdl. & Cham.	*Cinara* (*Cinara*) *pini*
92	*Pinus sylvestris* L.	*Cinara* (*Cinara*) *pinea*
93	*Pinus sylvestris* L. ‘Beuvronensis’	*Cinara* (*Cinara*) *pilosa*
94	*Polyspora* sp.	*Aphis* (*Aphis*) *fabae*
95	*Populus balsamifera* L.	*Pemphigus spyrothecae*, *Pterocomma populeum*
96	*Populus* × *canadensis* Moench	*Chaitophorus leucomelas*, *Pterocomma populeum*
97	*Populus* × *canescens* (Ait.) Sm.	*Chaitophorus populeti*
98	*Populus incrassata* Dode	*Pterocomma populeum*
99	*Populus grandidentata* Michx.	*Chaitophorus leucomelas*, *Pterocomma populeum*
100	*Populus nigra* L.	*Chaitophorus leucomelas*, *Pterocomma populeum*
101	*Populus nigra* L. subsp. *betulifolia* (Pursh) W. Wettst. ex Buttler & Hand	*Thecabius affinis*, *Chaitophorus leucomelas*, *Pterocomma populeum*
102	*Primula* sec. Proliferae	*Myzus* (*Myzus*) *ornatus*
103	*Primula* sp.	*Myzus* (*Myzus*) *ornatus*
104	*Prunus serrulata* Lindl. ‘Amanogawa’	*Myzus* (*Myzus*) *cerasi*
105	*Prunus* × *yedoensis* Matsum.	*Myzus* (*Nectarosiphon*) *persicae*
106	*Pseudosasa japonica* (Siebold & Zucc. ex Steud.) Makino ex Nakai	*Takecallis arundicolens*
107	*Pyrus* sp.	*Melanaphis pyraria*
108	*Rosa* ‘Jacques Cartier’	*Macrosiphum* (*Macrosiphum*) *rosae*
109	*Rosa* ‘Tuscany’	*Macrosiphum* (*Macrosiphum*) *rosae*
110	*Rosa* sp.	*Macrosiphum* (*Macrosiphum*) *rosae*, *Maculolachnus submacula*
111	*Quercus cornelius-mulleri* Nixon & K. P. Steele	*Lachnus pallipes*
112	*Quercus chenii* Nakai	*Thelaxes dryophila*
113	*Quercus dentata* Thunb.	*Phylloxera glabra*
114	*Quercus faginea* Lam.	*Lachnus roboris*
115	*Quercus falcata* Michx.	*Lachnus roboris*
116	*Quercus germana* Schltdl. & Cham.	*Thelaxes dryophila*
117	*Quercus* × *hispanica* Lam. ‘Lucombeana'	*Tuberculatus* (*Tuberculoides*) *annulatus*
118	*Quercus ilex* L.	*Thelaxes suberi*
119	*Quercus mongolica* Fisch. ex Ledeb.	*Myzocallis* (*Agrioaphis*) *castanicola*
120	*Quercus nigra* L.	*Lachnus roboris*
121	*Quercus palustris* Munchh.	*Lachnus pallipes*
122	*Quercus pontica* K. Koch	*Lachnus roboris*
123	*Quercus robur* L.	*Myzocallis* (*Agrioaphis*) *castanicola*, *Myzocallis* (*Myzocallis*) *boerneri*, *Tuberculatus* (*Tuberculatus*) *querceus*, *Tuberculatus* (*Tuberculoides*) *annulatus*
124	*Quercus rugosa* Née	*Thelaxes suberi*
125	*Quercus* × *sargentii* ‘Thomas’ Rehder	*Lachnus roboris*
126	*Quercus* sp.	*Lachnus roboris*
127	*Rheum palmatum* L.	*Aphis* (*Aphis*) *fabae*
128	*Rheum rhabarbarum* L.	*Aphis* (*Aphis*) *fabae*
129	*Rhododendron* ‘Golden Sunset’	*Illinoia (Masonaphis*) *lambersi*
130	*Rhododendron* sp.	*Aphis* (*Aphis*) *spiraecola*, *Ericolophium holsti*
131	*Ribes nigrum* L.	*Cryptomyzus* (*Cryptomyzus*) *ribis*
132	*Ribes orientale* Desf.	*Cryptomyzus* (*Cryptomyzus*) *korschelti*
133	*Ribes* sp.	*Aphis* (*Bursaphis*) *grossulariae*
134	*Rudbeckia* sp.	*Aphis* (*Aphis*) *fabae*
135	*Salix aegyptiaca* L.	*Aphis* (*Aphis*) *farinosa*
136	*Salix* × *fragilis* L.	*Chaitophorus salijaponicus niger*
137	*Salix lasiolepis* Benth.	*Chaitophorus horii beuthani*, *Pterocomma pilosum*
138	*Salix myrsinifolia* Salisb.	*Chaitophorus vitellinae*
139	*Salix prolixa* Andersson	*Aphis* (*Aphis*) *farinosa*
140	*Sasa palmata* (Burb.) E.G. Camus	*Takecallis arundinariae*, *Takecallis taiwanus*
141	*Saurauia napaulensis* DC.	*Myzus (Myzus) ornatus*
142	*Sedum telephium* L.	*Aphis* (*Aphis*) *sedi*, *Macrosiphum* (*Macrosiphum*) *hellebori*
143	*Sequoia sempervirens* (D. Don) Endl.	*Illinoia* (*Illinoia*) *morrisoni*
144	*Silybum marianum* (L.) Gaertn.	*Aphis* (*Aphis*) *fabae*
144	*Skimia* sp.	*Macrosiphum* (*Macrosiphum*) *euphorbiae*
146	*Solanum lycopersicum* L.	*Macrosiphum* (*Macrosiphum*) *euphorbiae*
147	*Solanum tuberosum* L.	*Aphis* (*Aphis*) *fabae*
148	*Tilia tomentosa* Moench	*Eucallipterus tiliae*
149	*Verbascum densiflorum* Bertol.	*Aphis* (*Aphis*) *verbasci*
150	*Viburnum farreri* Stearn	*Aphis* (*Aphis*) *fabae*
151	*Viburnum* sp.	*Aphis* (*Aphis*) *fabae*
152	*Vicia faba* L.	*Aphis* (*Aphis*) *fabae*
153	*Wahlenbergia angustifolia* (Roxb.) A. DC.	*Macrosiphum (Macrosiphum*) *euphorbiae*
154	*Yucca glauca* Nutt.	*Aphis* (*Aphis*) *fabae*
155	*Yucca* sp.	*Aphis* (*Aphis*) *fabae*, *Macrosiphum (Macrosiphum*) *euphorbiae*

The list of all collected species is presented in Table 2 and the Supplementary Material. In the Supplementary Material Aphidomorpha species were listed in systematic category alphabetically and sampling data for each aphid species include: locality, host plant, date and the unique sample number.

**Table 2:** Aphidomorpha collected during Eastop’s (1962, 1965) and the present (2017) study in the Royal Botanic Gardens, Kew.

No.	Taxon	Eastop		2017
		1962	1965	
	ADELGIDAE: ADELGINAE			
1	*Adelges laricis* Vallot, 1836			+
	PHYLLOXERIDAE: PHYLLOXERINAE			
2	*Phylloxera glabra* (von Heyden, 1837)			+
	APHIDIDAE: ERIOSOMATINAE			
3	*Eriosoma patchiae patchiae* (Bӧrner & Blunck, 1916)	+	+	
4	*Eriosoma patchiae lanuginosum* (Hartig, 1839)		+	
5	*Pemphigus bursarius* (Linnaeus, 1758)*	+		
6	*Pemphigus spyrothecae* Passerini, 1856	+		+
7	*Thecabius affinis* (Kaltenbach, 1843)*		+	+
	APHIDIDAE: ANOECIINAE			
8	*Anoecia corni* (Fabricius, 1775)*	+		+
	APHIDIDAE:THELAXINAE			
9	*Glyphina betulae* (Linnaeus, 1758)		+	
10	*Thelaxes dryophila* (Schrank, 1801)*	+	+	+
11	*Thelaxes suberi* (Del Guercio, 1911)		+	+
	APHIDIDAE: MINDARINAE			
12	*Mindarus abietinus* Koch, 1857	+		+
	APHIDIDAE: DREPANOSIPHINAE			
13	*Drepanosiphum platanoidis* (Schrank, 1801)*	_+_		+
	APHIDIDAE:PHYLLAPHIDINAE			
14	*Phyllaphis fagi* Linnaeus, 1767*	+		+
	APHIDIDAE:CALAPHIDINAE: Calaphidini			
15	*Betulaphis quadrituberculata* (Kaltenbach, 1843)		+	+
16	*Calaphis flava* Mordvilko, 1928*	+	+	+
17	*Callipterinella calliptera* (Hartig, 1841)			+
18	*Callipterinella minutissima* (Stroyan, 1953)		+	
19	*Clethrobius comes* (Walker, 1848)			+
20	*Euceraphis betulae* (Koch, 1855)			+
21	*Euceraphis punctipennis* (Zetterstedt, 1828)*	+	+	
22	*Monaphis antennata* (Kaltenbach, 1843)		+	+
23	*Symydobius oblongus* (Von Heyden, 1837)			+
	APHIDIDAE:CALAPHIDINAE: Panaphidini			
24	**!** *Chromaphis juglandicola* (Kaltenbach, 1843)*		+	+
25	*Eucallipterus tiliae* (Linnaeus, 1758)*	+		+
26	*Myzocallis* (*Agrioaphis*) *castanicola* Baker, 1917*	+		+
27	*Myzocallis* (*Myzocallis*) *boerneri* Stroyan, 1957	+		+
28	*Myzocallis* (*Myzocallis*) *carpini* (Koch, 1855)		+	+
29	*Myzocallis* (*Myzocallis*) *coryli* (Goeze, 1778)*		+	+
30	*Myzocallis* (*Myzocallis*) *schreiberi* Hille Ris Lambers & Stroyan, 1959		+	
31	**!** *Panaphis juglandis* (Goeze, 1778)*		+	+
32	*Pterocallis* (*Pterocallis*) *alni* (De Geer, 1773)			+
33	*Pterocallis* (*Pterocallis*) *maculata* (von Heyden, 1837)			+
34	**!** *Takecallis arundicolens* (Clarke, 1903)		+	+
35	**!** *Takecallis arundinariae* (Essig, 1917)		+	+
36	**!** *Takecallis taiwanus* (Takahashi, 1926)		+	+
37	*Tuberculatus* (*Tuberculatus*) *querceus* (Kaltenbach, 1843)*	+		+
38	*Tuberculatus* (*Tuberculoides*) *annulatus* (Hartig, 1841)	+		+
	APHIDIDAE:SALTUSAPHIDINAE			
39	*Subsaltusaphis* sp.		+	
	APHIDIDAE:CHAITOPHORINAE: Chaitophorini			
40	*Chaitophorus capreae* (Mosley, 1841)*	+	+	
41	*Chaitophorus horii beuthani* (Bӧrner, 1950)			+
42	*Chaitophorus leucomelas* Koch, 1854*	+		+
43	*Chaitophorus populeti* (Panzer, 1804)			+
44	*Chaitophorus salijaponicus niger* Mordvilko, 1929	+		+
45	*Chaitophorus vitellinae* (Schrank, 1801)			+
46	*Periphyllus acericola* (Walker, 1848)			+
47	**!** *Periphyllus californiensis* (Shinji, 1917)	+		+
48	*Periphyllus hirticornis* (Walker, 1848)		+	+
49	*Periphyllus lyropictus* (Kessler, 1886)			+
50	*Periphyllus testudinaceus* (Fernie, 1852)*	+	+	+
	APHIDIDAE:CHAITOPHORINAE: Siphini			
51	*Caricosipha paniculatae* Bӧrner, 1939		+	
	APHIDIDAE:APHIDINAE: Aphidini			
52	*Aphis* (*Aphis*) *comosa* (Bӧrner, 1950)		+	
53	*Aphis* (*Aphis*) *craccae* Linnaeus, 1758	+		
54	*Aphis* (*Aphis*) *cytisorum sarothamni* Franssen, 1928		+	
55	*Aphis* (*Aphis*) *fabae* Scopoli, 1763*	+	+	+
56	*Aphis* (*Aphis*) *fabae solanella* Theobald, 1914	+		
57	*Aphis* (*Aphis*) *farinosa* Gmelin, 1790			+
58	*Aphis* (*Aphis*) *genistae* Scopoli, 1763	+		
59	**!** *Aphis* (*Aphis*) *gossypii* Glover, 1877			+
60	*Aphis* (*Aphis*) *ilicis* Kaltenbach, 1843	+		+
61	*Aphis* (*Aphis*) *nasturtii* Kaltenbach, 1843	+		
62	*Aphis* (*Aphis*) *newtoni* Theobald, 1927			+
63	*Aphis* (*Aphis*) *pomi* De Geer, 1773			+
64	*Aphis* (*Aphis*) *praeterita* Walker, 1849	+		
65	*Aphis* (*Aphis*) *salicariae* Koch, 1855	+		
66	*Aphis* (*Aphis*) *sedi* Kaltenbach, 1843			+
67	**!** *Aphis* (*Aphis*) *spiraecola* Patch, 1914			+
68	*Aphis* (*Aphis*) *verbasci* Schrank, 1801			+
69	*Aphis* (*Bursaphis*) *epilobiaria* Theobald, 1927	+		
70	*Aphis* (*Bursaphis*) *epilobii* Kaltenbach, 1843*	+		
71	*Aphis* (*Bursaphis*) *grossulariae* Kaltenbach, 1843			+
72	**!** *Aphis* (*Toxoptera*) *aurantii* Boyer de Fonscolombe, 1841			+
73	*Hyalopterus pruni* (Geoffroy, 1762)		+	+
74	*Melanaphis luzulella* (Hille Ris Lambers, 1947)	+		
75	*Rhopalosiphum nymphaeae* (Linnaeus, 1761)		+	+
76	*Rhopalosiphum oxyacanthae* (Schrank, 1801)*	+	+	
77	*Rhopalosiphum padi* (Linnaeus, 1758)	+	+	
78	*Schizaphis* (*Paraschizaphis*) *scirpi* (Passerini, 1874)		+	
	APHIDIDAE:APHIDINAE: Macrosiphini			
79	*Acyrthosiphon* (*Acyrthosiphon*) *loti* (Theobald, 1913)	+		
80	*Acyrthosiphon* (*Acyrthosiphon*) *malvae* (Mosley, 1841)*	+	+	+
81	*Acyrthosiphon* (*Acyrthosiphon*) *pisum* Harris, 1776*	+	+	+
82	*Aulacorthum solani* (Kaltenbach, 1843)*	+	+	
83	*Brachycaudus* (*Acaudus*) *lychnidis* (Linnaeus, 1758)*		+	+
84	*Brachycaudus* (*Brachycaudus*) *helichrysi* (Kaltenbach, 1843)*	+		
85	*Brachycaudus* (*Prunaphis*) *cardui* (Linnaeus, 1758)			+
86	*Brachycolus cucubali* (Passerini, 1863)	+		
87	*Brevicoryne brassicae* (Linnaeus, 1758)*	+		
88	*Capitophorus hippophaes* (Walker, 1852)	+	+	
89	*Capitophorus inulae* (Passerini, 1860)	+		
90	*Capitophorus pakansus* Hottes & Frison, 1931	+		
91	*Cavariella aegopodii* (Scopoli, 1763)*	+	+	+
92	*Cavariella archangelicae* (Scopoli, 1763)		+	
93	*Cavariella pastinacae* (Linnaeus, 1758)		+	
94	*Cavariella theobaldi* (Gillete & Bragg, 1918)	+	+	
95	*Ceruraphis eriophori* (Walker, 1848)		+	
96	**!** *Chaetosiphon* (*Pentatrichopus*) *fragaefolii* (Cockerell, 1901)			+
97	*Coloradoa achilleae* Hille Ris Lambers, 1939		+	
98	*Coloradoa tanacetina* (Walker, 1850)	+		
99	*Corylobium avellanae* (Schrank, 1801)*		+	+
100	*Cryptaphis poae* (Hardy, 1850)		+	
101	*Cryptomyzus* (*Cryptomyzus*) *korschelti* Bӧrner, 1938			+
102	*Cryptomyzus* (*Cryptomyzus*) *ribis* (Linnaeus, 1758)			+
103	*Delphiniobium junackianum* (Karsch, 1887)	+	+	
104	*Diuraphis* (*Holcaphis*) *holci* (Hille Ris Lambers, 1956)		+	
105	*Dysaphis* (*Dysaphis*) *apifolia* (Theobald, 1923)	+		
106	*Dysaphis* (*Dysaphis*) *tulipae* (Boyer de Fonscolombe, 1841)			+
107	*Dysaphis* (*Pomaphis*) *pyri* (Boyer de Fonscolombe, 1841)*	+	+	
108	*Dysaphis* (*Pomaphis*) *plantaginea* (Passerini, 1860)			+
109	*Elatobium abietinum* (Walker, 1849)*		+	
110	**!** *Ericolophium holsti* (Takahashi, 1935)			+
111	*Hyadaphis passerinii* (Del Guercio, 1911)			+
112	*Hyalopteroides humilis* (Walker, 1852)		+	
113	*Hyperomyzus* (*Hyperomyzus*) *lactucae* (Linnaeus, 1758)	+		
114	*Hyperomyzus* (*Hyperomyzus*) *lampsanae* (Bӧrner, 1932)	+		
115	*Hyperomyzus* (*Neonasonovia*) *picridis* (Bӧrner & Blunck, 1916)		+	
116	**!** *Illinoia* (*Illinoia*) *andromedae* (MacGillivray, 1953)	+		
117	**!** *Illinoia* (*Illinoia*) *goldmayrae* (Knowlton, 1938)	+	+	
118	**!** *Illinoia* (*Illinoia*) *liriodendri* (Monell, 1879)			+
119	**!** *Illinoia* (*Illinoia*) *morrisoni* (Swain, 1918)	+		+
120	**!** *Illinoia* (*Masonaphis*) *lambersi* (MacGillivray, 1960)			+
121	*Linosiphon galiophagum* (Wimshurst, 1923)		+	
122	*Liosomaphis berberidis* (Kaltenbach, 1843)*	+		
123	*Lipaphis* (*Lipaphis*) *erysimi* (Kaltenbach, 1843)	+		
124	*Longicaudus trirhodus* (Walker, 1849)	+		
125	*Macrosiphoniella* (*Macrosiphoniella*) *abrotani* (Walker, 1852)	+		
126	*Macrosiphoniella* (*Macrosiphoniella*) *absinthii* (Linnaeus, 1758)	+	+	+
127	*Macrosiphoniella* (*Macrosiphoniella*) *artemisiae* (Boyer de Fonscolombe, 1841)		+	
128	*Macrosiphoniella* (*Macrosiphoniella*) *millefolii* (De Geer, 1773)*	+		+
129	**!** *Macrosiphoniella* (*Macrosiphoniella*) *sanborni* (Gillette, 1908)	+		
130	*Macrosiphoniella* (*Macrosiphoniella*) *sejuncta* (Walker, 1848)		+	
131	*Macrosiphoniella* (*Macrosiphoniella*) *tapuskae* (Hottes & Frison, 1931)		+	
132	*Macrosiphoniella* (*Phalangomyzus*) *oblonga* (Mordvilko, 1901)	+	+	
133	**!** *Macrosiphum* (*Macrosiphum*) *albifrons* Essig, 1911			+
134	*Macrosiphum* (*Macrosiphum*) *cholodkovskyi* (Mordvilko, 1909)	+		
135	*Macrosiphum* (*Macrosiphum*) *daphnidis* Bӧrner, 1950	+		
136	**!** *Macrosiphum* (*Macrosiphum*) *euphorbiae* (Thomas, 1878)*	+	+	+
137	*Macrosiphum* (*Macrosiphum*) *euphorbiellum* Theobald, 1917			+
138	*Macrosiphum* (*Macrosiphum*) *funestum* (Macchiati, 1885)		+	
139	*Macrosiphum* (*Macrosiphum*) *hellebori* Theobald &Walton, 1923	+		+
140	*Macrosiphum* (*Macrosiphum*) *rosae* (Linnaeus, 1758)*	+		+
141	*Macrosiphum* (*Macrosiphum*) *stellariae* Theobald, 1913		+	
142	*Megoura viciae* Buckton, 1876		+	+
143	*Melanaphis pyraria* (Passerini, 1861)			+
144	*Metopeurum fuscoviride* Stroyan, 1950	+		
145	*Metopolophium* (*Metopolophium*) *dirhodum* (Walker, 1849)*	+		
146	*Myzaphis rosarum* (Kaltenbach, 1843)		+	
147	*Myzus* (*Myzus*) *cerasi* (Fabricius, 1775)			+
148	*Myzus* (*Myzus*) *lythri* (Schrank, 1801)	+		
149	**!** *Myzus* (*Myzus*) *ornatus* Laing, 1932	+	+	+
150	**!** *Myzus* (*Nectarosiphon*) *persicae* (Sulzer, 1776)*	+		+
151	**!** *Myzus* (*Sciamyzus*) *ascalonicus* Doncaster, 1946	+	+	
152	**!** *Myzus* (*Sciamyzus*) *cymbalariae* Stroyan, 1954	+		
153	*Nasonovia* (*Nasonovia*) *ribisnigri* (Mosley, 1841)*	+		
154	**!** *Neomyzus circumflexus* (Buckton, 1876)*	+		
155	**!** *Neotoxoptera formosana* (Takahashi, 1921)			+
156	*Ovatomyzus stachyos* Hille Ris Lambers, 1947		+	
157	*Ovatus* (*Ovatus*) *crataegarius* (Walker, 1850)	+		
158	*Ovatus* (*Ovatus*) *insitus* (Walker, 1849)	+		
159	*Pterocomma pilosum* Buckton, 1879	+		+
160	*Pterocomma populeum* (Kaltenbach, 1843)	+	+	+
161	*Pterocomma rufipes* (Hartig, 1841)		+	
162	*Sitobion* (*Sitobion*) *avenae* (Fabricius, 1775)*	+	+	
163	*Sitobion* (*Sitobion*) *fragariae* (Walker, 1848)*		+	
164	**!** *Sitobion* (*Sitobion*) *luteum* (Buckton, 1876)	+		
165	*Tubaphis ranunculina* (Walker, 1852)		+	
166	*Uroleucon* (*Uroleucon*) *achilleae* (Koch, 1855)*	+		
167	*Uroleucon* (*Uroleucon*) *cichorii* (Koch, 1855)	+		
168	*Vesiculaphis theobaldi* Takahashi, 1930		+	
169	! *Wahlgreniella arbuti* (Davidson, 1910)		+	
	LACHNINAE: Eulachnini			
170	**!** *Cinara* (*Cinara*) *cedri* Mimeur, 1936			+
171	*Cinara* (*Cinara*) *cuneomaculata* (Del Guercio, 1909)		+	
172	*Cinara* (*Cinara*) *pectinatae* (Nӧrdlinger, 1880)	+		
173	*Cinara* (*Cinara*) *piceae* (Panzer, 1800)			+
174	*Cinara* (*Cinara*) *pilicornis* (Hartig, 1841)*	+	+	
175	*Cinara* (*Cinara*) *pilosa* (Zetterstedt, 1940)			+
176	*Cinara* (*Cinara*) *pinea* (Mordvilko, 1895)*		+	+
177	*Cinara* (*Cinara*) *pini* (Linnaeus, 1758)			+
178	*Cinara* (*Cupressobium*) *juniperi* (De Geer, 1773)*	+		
179	*Cinara* (*Schizolachnus*) *pineti* (Fabricius, 1781)	+		+
180	*Eulachnus agilis* (Kaltenbach, 1843)*	+		
181	*Eulachnus brevipilosus* Bӧrner, 1940		+	
182	*Eulachnus rileyi* (Williams, 1911)	+		
	LACHNINAE: Lachnini			
183	*Lachnus pallipes* (Hartig, 1841)			+
184	*Lachnus roboris* (Linnaeus, 1758)			+
185	*Maculolachnus submacula* (Walker, 1848)			+
	LACHNINAE: Tuberolachnini			
186	*Tuberolachnus salignus* (Gmelin, 1790)	+		

An exclamation mark [!] beside the name denotes alien species; a star mark * indicates species listed by Laing (1920).

## Discussion

According to Botanic Gardens Conservation International (BGCI), the Royal Botanic Gardens, Kew includes globally significant *ex situ* plant collections, covering approximately a third of known plant diversity, world-class seed banks, glasshouses and tissue culture infrastructures. It remains an open question, whether the Aphidomorpha present in Kew should be treated as an element of its biodiversity or an element threatening this diversity.

Aphids are strictly associated with their host plants. The presence of the host plant determines the presence of aphids, so it can be expected that with the constant species composition of plants in Kew, species composition of aphids will also be constant over time.

Comparing both Eastops aphid lists^28,29^, it can be seen that in the following years the species composition of the aphids varied significantly, in terms of quantity. In 1962, 91 species were found, while in 1965 77 taxa. In both lists we find only 25 common species. In 1962, greater variation was also demonstrated in the level of subfamilies and genera and the number of alien and pest species. Macrosiphini was dominant in both lists (Table 3). Comparing the whole aphid fauna collected by Eastop^28,29^, and during the present study, it is worth underlining, that in four years of collection, Eastop identified 142 taxa. In comparison, collecting aphids within twelve days allowed for identification of 95 species. The first conclusion is that the Kew aphid fauna is still rich and in a relatively short time a large number of aphid samples can be collected. However, comparing Eastop’s lists of species and results of the current study only 50 taxa are found in common (28 species in 1962 and 27 species in 1965). In the 1960’s Eastop collected 90 taxa that were not recorded during the present study. At the same time, current research has provided information on 45 species not listed by Eastop (Tables 2 and 3). Most of these species are in general widespread and common, some of them were collected by Eastop in Kew district but outside the Garden^28,29^ and are not included in the Table 2. The differences in the number of collected taxa results rather from the time spent collecting aphids (four years versus twelve days), than other conditions. The research was conducted in June, convenient due to the biology of aphids (both monoecious or heteroecious species) for collecting these insects. An exception may be species that in the early summer do not appear, like *Tuberolachnus salignus* (Gmelin, 1790), or which finish their life cycle earlier, such as aphids of the genus *Glyphina*^33^, both of them listed by Eastop^28,29^. The exception may also apply to species for various reasons considered rare in the Britain aphidofauna^33,34^, such as *Callipterinella calliptera* (Hartig, 1841), *Clethrobius comes* (Walker, 1848), *Monaphis antennata* (Kaltenbach, 1843), *Pterocallis* (*Pterocallis*) *maculata* (von Heyden, 1837) or *Lachnus pallipes* (Hartig, 1841), found during this study. As Macrosiphini are dominant among species that were not common on both lists, the number of economically important species of aphids, capable of virus transmission, is twice as high in Eastop’s study, as in the current survey. However, the number of species of foreign origin found during present study is twice as high compared to Eastop’s lists. This is obvious, because at least five of these species have been found in Europe in recent decades (e.g. *Illinoia* (*M*.) *lambersi* in 1971, *Macrosiphum* (*M*.) *albifrons* in 1981 or *Illinoia* (*I*.) *liriodendri* in 1998)^12^. It is also worth emphasizing, that among 155 listed host plants, 23 are regarded as threatened according to the IUCN. Most of them have a very limited distribution and a restricted habitat in their native range. For example, *Wahlenbergia angustifolia* is endemic to the island of St Helena, listed as Vulnerable^35^, whereas *Abies pinsapo*, distributed in small areas of Spain and Morocco, is listed as Endangered^36^. Our research proves, that far away from their natural range, in favourable conditions, they can be also colonized by aphids. In the case of endemic or native plant species, in their natural range, this threat can be important^37^. In addition to sampling aphids from threatened species of host plants, they were also collected from one of Kew Gardens’ Heritage trees, *Quercus* × hispanica ‘Lucombeana’, which is believed to have been planted at Kew in 1773.

**Table 3:** The most important quantitative data resulting from the Eastops’ lists (1962, 1965) and current research (2017).

	Eastop 1962	Eastop 1965	2017 (present study)
Total number of taxa	91	77	94
Total number of taxa	142		
Total number of taxa	186		
Common number of taxa	50		
Taxonomic comparison			
Adelginae	0	0	1
Phylloxerinae	0	0	1
Eriosomatinae	3	3	2
Anoeciinae	1	0	1
Thelaxinae	1	3	2
Mindarinae	1	0	1
Drepanosiphinae	1	0	1
Phyllaphidinae	1	0	1
Calaphidinae	7	13	21
Saltusaphidinae	0	1	0
Chaitophorinae	5	4	10
Aphidinae/Aphidini	13	8	13
Aphidinae/Macrosiphini	51	41	31
Lachninae	7	4	9
Alien species			
	12	10	20
Common number of taxa	4		
Pest species			
	25	17	20
Common number of taxa	9		

In total, 191 species of aphids have been listed from Kew^28–32,38^, including the present study, which is almost 1/3 of species presented in the check-list of aphids in Britain^39^. Kew includes globally significant *ex situ* collections, covering approximately a third of known plant diversity. Therefore, it is not surprising, that due to the diversity of host plants from different parts of the world, the variety of aphids associated with them is so large and it will probably grow.

Aphid species are not evenly distributed within Europe. The number of alien species present in a country is significantly and positively correlated with the number of native species recorded in that country, and, to a lesser extent, with the number of local taxonomists. Great Britain, with 65 alien aphid species, is on the top of European countries with identified numbers of those species. Among them, 36 were a first European record and at least five of them were first detected in Kew. Most of those species (18) came from North America, ten from Temperate Asia, two from Africa or tropical/subtropical areas of the world, respectively, two from Asia (generally) and two are cryptogenic^12^. The first record of alien species in the British aphidofauna (and Europe as a whole) concerned *Eriosoma lanigerum* (Hausmann, 1802) recorded in 1787^40^. The newest record is the presence of *Ericolophium holsti*, trapped in 2011^13^. The detection of species over the years has also been interesting. In 18^th^ and 19^th^ centuries there were three species, in 20^th^ century 30 species (with the greatest number between 1950–1980 when 14 alien species were recorded, ten from North America) and in 21^st^ century three species have been found^12,13^.

The aphid fauna of Kew includes a significant number of non-native aphid species. In 1962 and 1965 Eastop listed 18 alien species (on subsequent lists twelve and ten species, respectively). Among them *Illinoia* (*I*.) *andromedae* (MacGillivray, 1953) and *Illinoia* (*I*.) *goldamaryae* (Knowlton, 1938) (both from North America), were known as a first record for Europe. Unfortunately, during our study, the presence of those species in Kew was not confirmed. The third known species, recorded by Eastop^28^ as new for Europe - *Illinoia* (*I*.) *morrisoni* (Swain, 1918), associated with *Sequoia sempervirens*, was collected during the present study from the young shoots of its host plant in the Redwood Grove, in the same location as 60 years ago. In Britain, since Eastop’s original find, this species has been found three times – in Scotland (2001, suction-trap), South Wales (2007 from the host-plant) and Kent (2014, from the host-plant)^33^. However, now is treated as common and widely distributed in Britain^33^. In Europe, this species was recorded from France^41^, Italy^42^ and Portugal^43^.

Our inventory study brings data on ten additional non-native species of aphids detected in Kew (Table 2), at least half of these are known to be expanding their range. The clear movement of the alien aphid species is visible in the example of *Neotoxoptera formosana* (the onion aphid). The onion aphid, a pest of wild and cultivated (especially commercial) *Allium* has been recorded on the following hosts: *Allium ascalonicum*, *A*. *cepa*, *A*. *chinense*, *A*. *fistulosum*, *A*. *porrum*, *A*. *sativum*, *A*. *schoenoprasum*, *A*. *tuberosum*, and others^44^. In Europe this Asian species, is known from France (first record in 1984^45^), Finland (first record in 1994 on onions imported from the Netherlands), Italy (first record in 2000 on chives, *A*. *schoenoprasum* grown under glasshouse conditions^46^, Germany (first record in 2006 on stored onions in Konstanz (Bruehl, pers. comm.) and in 2007 in two fields of chives^47^) and the Netherlands (first record in 1994 and in 2008 on chives from a garden centre^48^). In the UK, this pest was found in September 1999, on a stock of Welsh onions (*A*. *fistulosum*) growing in a plastic tub in the Model Vegetable Garden at RHS Wisley, Surrey. The following year, in May, the species was again detected at RHS Wisley. *N*. *formosana* does not usually occur in the UK, although winged form was trapped in 40 ft aerial suction traps at Kirton, Lincolnshire in May 2002 and from Silwood Park in October 2005^44^. Later, the species was detected in Fife, Scotland in 2008 and on an onion purchased at a supermarket in Inverness, Scotland in August 2013^33^. It has a narrow host range. However, now is widespread and well established in Britain^49^ and it represents a potential risk to the UK *Allium* industry, which since 1995/96 has averaged approximately 13,000 ha with a value of just under £100 million. It can transmit viruses that cause plant damage and stunting although it is not a very efficient vector^44,47^. During the present study, the species was collected from the shoot of *Allium nutans* in the Rock Gardens of Kew. It proved that *N*. *formosana* can establish in Britain not only on *Allium* crops but also on common, wild *Allium* spp. and could survive in a cool maritime climate such as the UK.

To a lesser extent, we can now witness the expanding range of another non-native species. *Ericolophium holsti* is an alien species, first recorded in 2011 as new to Europe, which has only been recorded in the UK in the Rothamsted Insect Survey’s suction-traps. A single winged specimen was trapped in 2011 at Ascot, Berkshire, subsequently in 2012 three were caught at Rothamsted, Harpenden 60 km away^13^. In 2014 four specimens were caught, one each at Warwick, Harpenden, Hertfordshire; Boston, Lincolnshire; and Starcross, (near Exeter), Devon. This species of Asiatic origin, associated with *Rhododendron* spp., was not found in the field in Britain^50^. During this study, for the first time, the species was collected from the shoots of cultivated *Rhododendron* spp. in the Rhododendron Dell of Kew. The species was observed on three individuals of the host-plant – mostly winged morphs, however on one plant a colony of winged, wingless and nymphs was observed. This is the first record of *E*. *holsti* found in the field in the UK on its host. It is worth noting, that the new location in Kew (field study) is the closest to its original place of collection in Ascot, a distance of about 35 km (suction-trap). As a novel alien species detected in Europe, it is difficult to predict the impact of *E*. *holsti* on its host-plants. There was a similar situation for *Cinara curvipes* (Patch, 1912), which was first recorded in 1999 in Kew^32^ and soon spread to other parts of the UK^51^. Its spread into continental Europe was also very quick, as the species was detected in 2001 in Germany and Serbia; in 2007 in Switzerland, Czech Republic, Slovakia and Slovenia; in 2013 in Hungary; in 2014 in Austria; and in 2015 in Poland. *C*. *curvipes* is able to infest native European coniferous trees and in some countries, has pest status^52,53^.

Thus, in Kew 30 species non-native for Europe have been listed so far^28,29,31,32^, including the present study, which is half of all known non-native species detected in the UK. The combination of factors like a large and diverse collections of plants, the majority of which are exotic in Kew, the short distance to airports (Heathrow airport <11 km), seaports (the Port of London ~16 km) and human population density (London), promotes both the settlement and the spread of species of foreign origin, but firstly the introduction^54^. In particular, potential hotspots of invasions such as airports, should be monitored as a priority to prevent new invasions from these species^55,56^ (e.g. at Heathrow, one of the world’s busiest airports close to Kew, various plants, including threatened ones, have been confiscated^57^). As Aphidomorpha are small insects, easily transported by air or with plant material, the number of introductions of aliens will probably increase, this is also linked to the continued expansion of the worldwide air transportation network^58^. Moreover, aphids are able to adapt to climate change faster than many other insect groups studied because of their low developmental threshold temperature and high intrinsic rate of increase^39^. Botanic gardens are not substitutes for study in natural areas but should be viewed as complementary. The plants are well identified thus making the identification of insects, even from exotic plants, easier. In particular, in the case of Aphidomorpha, which are mostly strictly associated with their host plants. The key factor is the prevention of an introduction of a non-native species. If prevention fails, then early detection and rapid response to remove the species becomes very important. It is easier to fight invasiveness if the discovery of the non-native species is made early^59^. With simple tools (short-term faunistic inventory of important insects) we achieved effective results. According to this, botanic gardens shouldn’t be the gateway for alien species, but instead the gateway to information on alien and invasive ones. Therefore, the database of such species (even in form of simple list) will help identify the scale and spatial pattern of invasive alien and pest species and can be used as a framework for considering indicators for early warning as well as a model for other studies.

## Material and Methods

### Collecting area

The Royal Botanic Gardens, Kew (Kew) are situated in the London Borough (district) of Richmond upon Thames, in southwest Greater London, United Kingdom, 51° 28′ 0.12″ N 0° 16′ 59.88″ W. Surveys reported here were carried out mostly in the collection of living plants grown unprotected outside in the Gardens, as well as a limited number in controlled conditions within glasshouses and nurseries on site. The abbreviations denote as follow: AColl. – *Acer* spp. collection; ARBN – Arboretum Nursery; BColl. – *Betula* spp. collection; PColl. – *Populus* spp. collection; QColl. – *Quercus* spp. collection; AG – Aquatic Garden; BG – Bamboo Garden; DG – Duke’s Garden; JG – Japanese Garden; L – Lake; MG – Mediterranean Garden; NO – near Orangery; P – Pond; PFB – Plant Family Beds; Pi – Pinetum; RD – Rhododendron Dell; RG – Rock Garden; RK – Royal Kitchen; ReG – Redwood Grove; RoG – Rose Garden; SVP – Student Vegetables Plot; TRON – Tropical Nursery. In the case of unlocalized records, the exact situation of the host plant was not specially noted. The source of map (Fig. 1) of collecting areas was the Gardens Development Unit, the Royal Botanic Gardens, Kew. The Fig. 1 was prepared using Corel Draw 17.1.0.572, 2014 Corel Corporation.

### Sampling procedure

The investigation was conducted over a period of twelve days, from 5th to 16th June 2017. The aphids were collected directly from the host plants with a fine hair brush and placed into Eppendorf tubes containing 70% and 98% ethanol. Location, sampling date and host plant name were recorded on the labels placed onto the tubes.

### Species identification

Adult wingless (apt. viv.) or winged (al. viv.) females (or aestivating larvae in the case of the genus *Periphyllus* van der Hoeven) were slide mounted using the method of Kanturski and Wieczorek^60^ and identified to species level. The slides were examined using a Nikon Ni-U light microscope. Names and classification follow Nieto Nafría and Favret^61^, with the exception of the taxonomic position of all the former Pterocommatinae, which have been placed in the tribe Macrosiphini. Samples were identified by K. Wieczorek based on morphological diagnostic features using standard literature-based keys^49,62–71^. Only small amount of samples were not identified as the immature generations (larvae or nymphs) were collected. These samples were not included into the list of species. The lists of alien Aphididae in Europe^12^ were used to identify the alien species. An exclamation mark [!] beside the name denotes those species. Aliens are treated as species with native ranges outside Europe. Pest status was given according to Blackman and Eastop^1^. The aphid material is deposited in the collection of the Department of Zoology, University of Silesia, Katowice, Poland (DZUS) and will be subsequently digitalized. Voucher specimens for collected samples in 98% ethanol are deposited in the Lab-based Collections Royal Botanic Gardens, Kew, London, UK. The sources for the botanical nomenclature was the International Plant Names Index^72^.

## Supplementary information

Supplementary Information.

## References

[1] Blackman, R. L. & Eastop, V. F. Aphids on the World’s Crops. An Identification and Information Guide. Second Edition 1–466 (The Natural History Museum, 2000).

[2] van Emden, F. & Harrington, R. Aphids as Crop Pests 1–717 (CABI, 2007).

[3] Saguez, J., Giordanengo, P. & Vincent, C. Chapter 3. Aphids as major potato pests. (eds Giordanengo, P., Vincent, Ch. & Alyokhin, A.) Insect Pests of Potato: global perspectives on biology and management. 31–63 (Elsevier, 2012).

[4] Tilmon KJ (2011). Biology of the Soybean Aphid, Aphis glycines (Hemiptera: Aphididae) in the United States. J Integr Pest Manag..

[5] Michaud, J. P. Chapter 5. Implications of climate change for cereal aphids on the Great Plains of North America. (eds Kindlmann, P., Dixon, A. F. G. & Michaud, J. P.). Aphids biodiversity under environmental change. 69–91(Springer, 2010).

[6] Bosque-Perez NA, Eigenbrode SD (2011). The influence of virus-induced changes in plants on aphid vectors: Insights from luteovirus pathosystem. Virus Research.

[7] Bass C (2014). The evolution of insecticide resistance in the peach potato aphid, *Myzus persicae*. Insect Biochem Mol Biol..

[8] Skvarla MJ (2017). An Update to the Adventive Aphids (Hemiptera: Aphidoidea) of America North of Mexico, with Notes on Intercepted Species. Proc. Entomol. Soc. Wash..

[9] CABI, *Diuraphis noxia*. In: Invasive Species Compendium. Wallingford, UK: CAB International. www.cabi.org/isc (2018).

[10] Lee Y, Kim S, Lee S (2018). A first record of three aphid pests (Aphididae: Calaphidinae) on walnut in Korea. Journal of Asia-Pacific Biodiversity..

[11] Skvarla Michael J., Halbert Susan E., Foottit Robert G., Jensen Andrew S., Maw Eric, Miller Gary L. (2017). An Update to the Adventive Aphids (Hemiptera: Aphidoidea) of America North of Mexico, with Notes on Intercepted Species. Proceedings of the Entomological Society of Washington.

[12] Couer d’acier, A., Pérez Hidalgo, N. & Petrović-Obradović, O. Chapter 9.2. Aphids (Hemiptera, Aphididae). (eds Roques *et al*.) Alien terrestrial arthropods of Europe. *BioRisk* **4****(****1****)**, 435–474 (2010).

[13] Eastop VF, Blackman RL, Taylor MS (2012). Ericolophium holsti (Hemiptera: Aphididae), a new rhododendron-feeding aphid for Britain and Europe. Br. J. Ent. Nat. Hist..

[14] Pellizari G, Frigimelica G (2014). First record and establishment of *Tuberocephalus* (*Trichosiphoniella*) *tianmushanensis* Zang (Hemiptera, Aphididae) on ornamental cherry trees in Italy. J. entomological acarological Research.

[15] Roberts P, Ruberson J (2008). Cotton insects. Summary of Losses from Insect Damage and Cost of Control in Georgia 2006. Miscellaneous Publications.

[16] Kular JS, Kumar S (2011). Quantification of avoidable yield losses in oilseed Brassica caused by insect pests. J Plant Prot Res..

[17] Khan, A. M., Khan, A. A., Afzal, M. & Iqbal, M. S. Wheat Crop Yield Losses Caused by the Aphids Infestation. *J Biofertil Biopestici* **3****(****4****)**, 2–7, 10.4172/2155-6202.1000122 2011 (2012).

[18] Wieczorek K, Bugaj-Nawrocka A (2014). Invasive aphids of the tribe Siphini: a model of potentially suitable ecological niches. Agr Forest Entomol..

[19] Valenzuela I, Hoffmann AA (2015). Effects of aphid feeding and associated virus injury on grain crops in Australia. Aust Entomol..

[20] Lazzarotto CM, Lazzari SMN, Penteado SRC (2015). Feeding behavior in two exotic aphid species on their original hosts in a new invaded area. Neotrop Entomol.

[21] Pèrez Hidalgo N (2000). Dos especies de *Cerataphis* (Hemiptera: Aphididae: Hormaphidinae) introducidas en las Islas Canarias. Biol. San. Veg. Plagas.

[22] Heywood VH (2011). The role of botanic gardens as resource and introduction centres in the face of global change. Biodivers. Conserv..

[23] Galera H, Sudnik-Wójcikowska B (2010). Central European Botanic Gardens as centres of dispersal of alien plants. Acta Soc Bot Pol.

[24] Hulme PE (2011). Addressing to threat to biodiversity from botanic gardens. Trends Ecol. Evol..

[25] Hulme PE (2015). Resolving whether botanic gardens are on the road to conservation or a pathway for plant invasions. Conserv Biol.

[26] Ronse A (2011). ‘Botanical garden escapes’ in the National Botanic Garden of Belgium. Scr Bot Belg..

[27] Baylis HA (1920). Additions to the Wild Fauna and Flora of the Royal Botanic Gardens, Kew XV. Bulletin of Miscellaneous Information.

[28] Eastop VF (1962). Additions to the Wild Fauna and Flora of the Royal Botanic Gardens, Kew. A Contribution to the Aphid Fauna. Kew Bulletin.

[29] Eastop VF (1965). Additions to the Wild Fauna and Flora of the Royal Botanic Gardens, Kew. A Second Contribution to the Aphid Fauna. Kew Bulletin.

[30] Prior RNB (1971). Some notes on new or uncommon aphids recently found in Britain. Zool. J. of the Linn. Soc..

[31] Polaszek, A. Comparative anatomy of the male aphid reproductive system. *Unpublished Ph*.*D*. *Thesis* (1987).

[32] Martin JH (2000). Two new British aphid introductions in 1999, in the context of the other additions over the preceding thirty years (Sternorrhyncha: Aphidoidea). Entomol. Gaz..

[33] http://influentialpoints.com/Index.htm (2018).

[34] Stroyan, H. L. G. Homoptera: Aphidoidea (Part) – Chaitophoridae and Callaphididae. Handbooks for the identification of British insects 2(4a). *Royal Entomological Society*, *London* (1977).

[35] Lambdon, P. W. & Ellick, S. *Wahlenbergia angustifolia*. The IUCN Red List of Threatened Species 2016: e.T43988A67371447., 10.2305/IUCN.UK.2016-1.RLTS.T43988A67371447.en (2016).

[36] Arista, A., Alaoui, M. L., Knees, S. & Gardner, M. *Abies pinsapo*. The IUCN Red List of Threatened Species 2011: e.T42295A10679577., 10.2305/IUCN.UK.2011-2.RLTS.T42295A10679577.en (2011).

[37] Hullè M (2012). *Myzus ascalonicus*, an aphid recently introduced to Sub-Antarctic Islands, Prefers Native to Exotic Host-Plants. Environ Entomol.

[38] Luker S (2011). *Crypturaphis grassi* (Sternorrhyncha: Aphididae): First records for Cornwall. Br. J. Ent. Nat. Hist..

[39] Bell JR (2015). Long-term phenological trends, species accumulation rates, aphid traits and climate: five decades of change in migrating aphids. J Anim Ecol.

[40] Balachowsky, A. & Mesni L. *Les insects nuisibles aux plantes cultivees* 1–1921 (Mery L. 1935).

[41] Rabasse JM (2005). On the presence in Europe of two *Illinoia* aphids of North American origin (Homoptera, Aphidoidea). Boll Zool Agrar Bachic.

[42] Coceano PG, Petrovic-Obradovic O (2006). New aphid species for Italy caught by suction trap. Phytoparasitica.

[43] Rodrigues P, Ilharco FA, Silva EB, Franco JC (2006). Interactions between ground cover management, hedges and aphids in lemon orchards. Bulletin OILB/SROP.

[44] MacLeod, A. CSL pest risk analysis for *Neotoxoptera formosana* 1–2 (The food and Environment Research Agency, Plant Health Risk Management, 2007).

[45] Leclant, F. Les pucerons des plantes cultivées. Clefs d’identification. II Cultures maraîchères. 1–98 (Acta/INRA, 1999).

[46] Barbagallo S, Ciampolini M (2000). The onion aphid, *Neotoxoptera formosana* (Takahashi), detected in Italy. Boll Zool Agrar Bachic..

[47] Schrameyer K (2008). Blattläuse auch bei Allium-Arten. Gemüse.

[48] Piron PGM (2010). Appearance of *Neotoxoptera formosana* (Homiptera: Aphididae) in The Netherlands. Entomol. Ber..

[49] Blackman, R. L. Aphids - Aphidinae (Macrosiphini). Handbook for identification of British insects 1–414 (Royal Entomological Society, 2010).

[50] Anon. New Rhododendron aphids on the Move. http://resources.rothamsted.ac.uk/insect-survey-resources/new-rhododendron-aphids-move (2017)

[51] Baker EA (2009). Observations of aphids (Aphidoidea) new to Wales. Br. J. Ent. Nat. Hist..

[52] Jurc M, Poljaković-Pajnik, Jurc D (2009). The first record of *Cinara curvipes* (Patch, 1912) (Homoptera, Aphididae) in Slovenia and its possible impact. Zbornik gozdarstva in lesarstva.

[53] Hałaj R, Osiadacz B (2015). On foreign land: the conquest of Europe by *Cinara curvipes* (Patch, 1912). Dtsch. entomol. Z..

[54] Wylie, F. S. & Speight M. R. Insect Pests in Tropical Forestry. CABI (2012).

[55] Inghilesi AF (2013). Alien insects in Italy: Comparing patterns from the regional to European level. J Insect Sci..

[56] Toral-Grande V (2017). Alien species pathways to the Galapagos Islands, Ecuador. PLoS One.

[57] https://stateoftheworldsplants.org/2017/report/SOTWP_2017_12_plant_conservation_policies_and_international_trade.pdf.

[58] Bellard C (2016). Major drivers of invasion risks throughout the world. Ecosphere.

[59] *Committee* for *Environmental Protection* (*CEP*). Non-native Species Manual Secretariat of the Antarctic Treaty 1–47 (Edition 2017).

[60] Kanturski, M. & Wieczorek, K. Mszyce (Hemiptera, Aphidoidea) jako grupa modelowa w badaniach zoocenologicznych 123–131 [In Polish] (ed. Kuczera, M. Nowe trendy w naukach przyrodniczych Creative Science, 2012).

[61] Nieto Nafría J.M. *et al*. Register of genus-group taxa of Aphidoidea. *In* Nieto Nafría, J. M. and Favret, C. (eds.), Registers of Family-group and genus-group taxa of Aphidoidea (Hemiptera Sternorrhyncha) 81–404 (Universitad de León, 2011).

[62] Blackman, R. L. & Eastop, V. F. Aphids on the World’s Trees. An Identification and Information Guide 1–1016 (CABI, 1994).

[63] Blackman, R. L. & Eastop V. F. Aphids on the World’s Herbaceous Plants and Shrubs 1–1439 (The Natural History Museum, 2006).

[64] Blackman, R. L. & Eastop, V. F. Aphids of the World’s Plants: An Online Identification and Information Guide. http://www.aphidsonworldsplants.info (2017).

[65] Heie, O.E. The Aphidoidea (Hemiptera) of Fennoscandia and Denmark. I. 1–236 (Scandinavian Science Press, 1980).

[66] Heie, O.E. The Aphidoidea (Hemiptera) of Fennoscandia and Denmark. II. 1–175 (Scandinavian Science Press, 1982).

[67] Heie, O.E. The Aphidoidea (Hemiptera) of Fennoscandia and Denmark. III. 1–314 (E.J. Brill, 1986).

[68] Heie, O.E. The Aphidoidea (Hemiptera) of Fennoscandia and Denmark. IV. 1–189 (E.J. Brill, 1992).

[69] Heie, O.E. The Aphidoidea (Hemiptera) of Fennoscandia and Denmark. V. 1–239 (E.J. Brill, 1994).

[70] Heie, O.E. The Aphidoidea (Hemiptera) of Fennoscandia and Denmark. VI. 1–217 (E.J. Brill, 1995).

[71] Junkiert Ł, Wieczorek K, Wojciechowski W (2011). Diagnostic characters of the species of the genus Periphyllus van der Hoeven, 1863 (Hemiptera, Aphidoidea: Chaitophorinae) recorded in Poland. Aphids and other Hemipterous Insects.

[72] Kew Names and Taxonomic Backbone. The International Plant Names Index and World Checklist of Selected Plant Families 2019. Published on the Internet at http://www.ipni.org and http://apps.kew.org/wcsp/.

