# Supplementary material for: The composition of the aphid fauna (Insecta, Hemiptera) of the Royal Botanic Gardens, Kew
Source: Sci Rep. 2019 Jul 10;9:10000. doi: 10.1038/s41598-019-46441-z (PMC6620339; doi:10.1038/s41598-019-46441-z)
Supplement: Supplementary file 1 — Supplementary Information. [file 41598_2019_46441_MOESM1_ESM.pdf]

The composition of the aphid fauna (Insecta, Hemiptera) of the Royal Botanic Gardens, Kew

Karina Wieczorek, Tim K. Fulcher and Dominik Chłond

Supplementary Information.

Supplementary Information. The list of species collected in the Royal Botanic Gardens, Kew. Aphidomorpha species are listed in systematic category alphabetically and sampling data for each aphid species include: locality, host plant, date and the unique sample number.

ADELGIDAE: ADELGINAE

1. *Adelges laricis* Vallot, 1836: London, Kew Garden, UK, 14.6.2017, *Picea* sp., Pi, 1 al. viv., K. Wieczorek, DZUS KG14/6/17_171.

PHYLLOXERIDAE: PHYLLOXERINAE

2. *Phylloxera glabra* (von Heyden, 1837): London, Kew Garden, UK, 7.6.2017, *Quercus dentata*, QColl., 3 apt. viv., K. Wieczorek, DZUS KG7/6/17_26.

APHIDIDAE: ERIOSOMATINAE

3. *Pemphigus spyrothecae* Passerini, 1856: London, Kew Garden, UK, 15.6.2017, *Populus balsamifera*, PColl., 2 apt. viv., K. Wieczorek, DZUS KG15/6/17_187.

4. *Thecabius affinis* (Kaltenbach, 1843): London, Kew Garden, UK, 12.6.2017, *Populus nigra betulifolia*, ARBN, 2 apt. viv., K. Wieczorek, DZUS KG12/6/17_129.

APHIDIDAE: ANOECIINAE

5. *Anoecia corni* (Fabricius, 1775): London, Kew Garden, UK, 13.6.2017, *Cornus* sp., L, 2 apt. viv., K. Wieczorek, DZUS KG13/6/17_156.

APHIDIDAE:THELAXINAE

6. *Thelaxes dryophila* (Schrank, 1801): London, Kew Garden, UK, 8.6.2017, *Quercus chenii*, QColl., 3 apt. viv., K. Wieczorek, DZUS KG8/6/17_33, 3 apt. viv., DZUS KG8/6/17_34; London, Kew Garden, UK, 16.6.2017, *Quercus germana*, AG, 3 apt. viv., K. Wieczorek, DZUS KG16/6/17_220.

7. *Thelaxes suberi* (Del Guercio, 1911): London, Kew Garden, UK, 7.6.2017, *Quercus ilex*, QColl., 3 apt. viv., 1 al. viv., K. Wieczorek, DZUS KG7/6/17_27; London, Kew Garden, UK, 15.6.2017, *Quercus rugosa*, near Play Ground, 3 apt. viv., K. Wieczorek, DZUS KG15/6/17_185.

APHIDIDAE: MINDARINAE

8. *Mindarus abietinus* Koch, 1857: London, Kew Garden, UK, 14.6.2017, *Abies pinsapo*, Pi, 2 al. viv., K. Wieczorek, DZUS KG14/6/17_172.

APHIDIDAE: DREPANOSIPHINAE

9. *Drepanosiphum platanoidis* (Schrank, 1801): London, Kew Garden, UK, 13.6.2017, *Acer oblongum*, 3 al. viv., K. Wieczorek, DZUS KG13/6/17_149; London, Kew Garden, UK,

14.6.2017, *Acer heldreichii* subsp. *trautvetteri* , AColl., 2 al. viv., K. Wieczorek, DZUS KG14/6/17_173; *Acer pseudoplatanus*, 1 al. viv., DZUS KG14/6/17_179.

APHIDIDAE: PHYLLAPHIDINAE

10. *Phyllaphis fagi* Linnaeus, 1767: London, Kew Garden, UK, 7.6.2017, *Fagus sylvatica*, 2 al. viv., K. Wieczorek, DZUS KG7/6/17_16; London, Kew Garden, UK, 7.6.2017, *Liriodendron chinense*, accidentally, 1 al. viv., K. Wieczorek, DZUS KG7/6/17_22; London, Kew Garden, UK, 7.6.2017, *Quercus mongolica*, accidentally, QColl., 2 al. viv., K. Wieczorek, DZUS KG7/6/17_28; London, Kew Garden, UK, 9.6.2017, *Fagus sylvatica* 'Tricolor', ARBN, 1 al. viv., K. Wieczorek, DZUS KG9/6/17_55.

APHIDIDAE: CALAPHIDINAE: Calaphidini

11. *Betulaphis quadrituberculata* (Kaltenbach, 1843): London, Kew Garden, UK, 12.6.2017, *Betula utilis* var. *prattii*, BColl., 1 al. viv., K. Wieczorek, DZUS KG12/6/17_122.

12. *Calaphis flava* Mordvilko, 1928: London, Kew Garden, UK, 8.6.2017, *Betula ermanii*, BColl., 2 al. viv., K. Wieczorek, DZUS KG8/6/17_44; London, Kew Garden, UK, 12.6.2017, *Betula utilis* var. *prattii*, BColl., 1 al. viv., K. Wieczorek, DZUS KG12/6/17_123b; London, Kew Garden, UK, 16.6.2017, *Betula dahurica*, 1 apt. viv., K. Wieczorek, DZUS KG16/6/17_218.

13. *Callipterinella calliptera* (Hartig, 1841): London, Kew Garden, UK, 8.6.2017, *Betula grossa*, BColl., 2 al. viv., K. Wieczorek, DZUS KG8/6/17_46.

14. *Clethrobius comes* (Walker, 1848): London, Kew Garden, UK, 8.6.2017, *Betula platyphylla*, BColl., 1 al. viv., K. Wieczorek, DZUS KG8/6/17_45.

15. *Euceraphis betulae* (Koch, 1855): London, Kew Garden, UK, 7.6.2017, *Betula pendula*, BColl., 1 al. viv., K. Wieczorek, DZUS KG7/6/17_10; London, Kew Garden, UK, 7.6.2017, *Betula szechuanica*, BColl., 2 al. viv., K. Wieczorek, DZUS KG7/6/17_11.

16. *Monaphis antennata* (Kaltenbach, 1843): London, Kew Garden, UK, 12.6.2017, *Betula utilis* var. *prattii*, BColl., 1 al. viv., K. Wieczorek, DZUS KG12/6/17_123a.

17. *Symydobius oblongus* (von Heyden, 1837): London, Kew Garden, UK, 8.6.2017, *Betula utilis*, BColl., 1 apt. viv., 1 al. viv., K. Wieczorek, DZUS KG8/6/17_40; London, Kew Garden, UK, 8.6.2017, *Betula litwinowii*, BColl., 2 apt. viv., 1 al. viv., K. Wieczorek, DZUS KG8/6/17_47; London, Kew Garden, UK, 16.6.2017, *Betula albosinensis*, AG, 1 apt. viv., 1 al. viv., K. Wieczorek, DZUS KG16/6/17_221.

APHIDIDAE:CALAPHIDINAE: Panaphidini

18. *Chromaphis juglandicola* (Kaltenbach, 1843): London, Kew Garden, UK, 10.6.2017, *Juglans regia*, 1 al. viv., K. Wieczorek, DZUS KG10/6/17_79.

19. *Eucallipterus tiliae* (Linnaeus, 1758): London, Kew Garden, UK, 7.6.2017, *Tilia tomentosa*, 1 al. viv., K. Wieczorek, DZUS KG7/6/17_12.
20. *Myzocallis* (*Agrioaphis*) *castanicola* Baker, 1917: London, Kew Garden, UK, 6.6.2017, *Quercus robur*, QColl., 2 al. viv., K. Wieczorek, DZUS KG6/6/17_9; London, Kew Garden, UK, 6.6.2017, *Castanea sativa*, 2 al. viv., K. Wieczorek, DZUS KG6/6/17_13; London, Kew Garden, UK, 8.6.2017, *Quercus mongolica*, 2 al. viv., K. Wieczorek, DZUS KG8/6/17_31.
21. *Myzocallis* (*Myzocallis*) *boerneri* Stroyan, 1957: London, Kew Garden, UK, 7.6.2017, *Quercus robur*, QColl., 1 al. viv., K. Wieczorek, DZUS KG7/6/17_14.
22. *Myzocallis* (*Myzocallis*) *carpini* (Koch, 1855): London, Kew Garden, UK, 9.6.2017, *Carpinus cordata* var. *chinensis*, BG, 1 al. viv., K. Wieczorek, DZUS KG9/6/17_70.
23. *Myzocallis* (*Myzocallis*) *coryli* (Goeze, 1778): London, Kew Garden, UK, 7.6.2017, *Coryllus avellana*, QColl., 2 al. viv., K. Wieczorek, DZUS KG7/6/17_30.
24. *Panaphis juglandis* (Goeze, 1778): London, Kew Garden, UK, 10.6.2017, *Juglans regia*, 2 apt. viv., K. Wieczorek, DZUS KG10/6/17_80.
25. *Pterocallis* (*Pterocallis*) *alni* (De Geer, 1773): London, Kew Garden, UK, 13.6.2017, *Alnus rubra*, L, 2 al. viv., K. Wieczorek, DZUS KG13/6/17_153.
26. *Pterocallis* (*Pterocallis*) *maculata* (Von Heyden, 1837): London, Kew Garden, UK, 13.6.2017, *Alnus glutinosa*, L, 2 al. viv., K. Wieczorek, DZUS KG13/6/17_161.
27. *Takecallis arundicolens* (Clarke, 1903): London, Kew Garden, UK, 9.6.2017, *Pseudosasa japonica*, BG, 2 al. viv., K. Wieczorek, DZUS KG9/6/17_69.
28. *Takecallis arundinariae* (Essig, 1917): London, Kew Garden, UK, 9.6.2017, *Bambusa* sp., BG, 2 al. viv., K. Wieczorek, DZUS KG9/6/17_68a; *Phyllostachys aurea*, BG, 1 al. viv., DZUS KG9/6/17_68b; *Sasa palmata* f. *nebulosa*, BG, 2 al. viv., DZUS KG9/6/17_68c.
29. *Takecallis taiwanus* (Takahashi, 1926): London, Kew Garden, UK, 9.6.2017, *Sasa palmata* f. *nebulosa* , BG, 2 al. viv., K. Wieczorek, DZUS KG9/6/17_71.
30. *Tuberculatus* (*Tuberculatus*) *querceus* (Kaltenbach, 1843): London, Kew Garden, UK, 12.6.2017, *Quercus robur*, ARBN, 2 al. viv., K. Wieczorek, DZUS KG12/6/17_127.
31. *Tuberculatus* (*Tuberculoides*) *annulatus* (Hartig, 1841): London, Kew Garden, UK, 6.6.2017, *Quercus robur*, QColl., 3 al. viv., K. Wieczorek, DZUS KG6/6/17_8; London, Kew Garden, UK, 7.6.2017, *Quercus robur*, QColl., 3 al. viv., K. Wieczorek, DZUS KG7/6/17_15; London, Kew Garden, UK, 7.6.2017, *Quercus* x *hispanica* 'Lucombeana', 3 al. viv., K. Wieczorek, DZUS KG7/6/17_17.

APHIDIDAE:CHAITOPHORINAE: Chaitophorini

32. *Chaitophorus horii beuthani* (Bőrner, 1950): London, Kew Garden, UK, 13.6.2017, *Salix lasiolepis*, L, 2 apt. viv., K. Wieczorek, DZUS KG13/6/17_159.

33. *Chaitophorus leucomelas* Koch, 1854: London, Kew Garden, UK, 7.6.2017, *Populus nigra betulifolia*, PColl., 3 apt. viv., K. Wieczorek, DZUS KG7/6/17_20; London, Kew Garden, UK, 7.6.2017, *Populus grandidentata*, PColl., 3 apt. viv., K. Wieczorek, DZUS KG7/6/17_24; London, Kew Garden, UK, 12.6.2017, *Populus nigra betulifolia*, ARBN, 2 apt. viv., K. Wieczorek, DZUS KG12/6/17_128, 2 apt. viv., 1 al. viv., DZUS KG12/6/17_129; London, Kew Garden, UK, 15.6.2017, *Populus* x *canadensis*, PColl., 2 apt. viv., K. Wieczorek, DZUS KG15/6/17_186, *Populus rasumovskae*, PColl., 1 apt. viv., 1 al. viv., K. Wieczorek, DZUS KG15/6/17_189, *Populus rasumovskae*, PColl., 1 apt. viv., 1 al. viv., DZUS KG15/6/17_190, *Populus nigra*, 3 apt. viv., PColl., DZUS KG15/6/17_197.

34. *Chaitophorus populeti* (Panzer, 1804): London, Kew Garden, UK, 7.6.2017, *Populus canescens*, PColl., 2 apt. viv., K. Wieczorek, DZUS KG7/6/17_18.

35. *Chaitophorus salijaponicus niger* Mordvilko, 1929: London, Kew Garden, UK, 10.6.2017, *Salix* x *fragilis*, P, 2 apt. viv., K. Wieczorek, DZUS KG10/6/17_74.

36. *Chaitophorus vitellinae* (Schrank, 1801): London, Kew Garden, UK, 13.6.2017, *Salix myrsinifolia*, L, 2 apt. viv., K. Wieczorek, DZUS KG13/6/17_160.

37. *Periphyllus acericola* (Walker, 1848): London, Kew Garden, UK, 14.6.2017, *Acer heldreichii* subsp. *trautvetteri* , AColl., 2 aestivating larvae, K. Wieczorek, DZUS KG14/6/17_174; *Acer griseum*, 2 aestivating larvae, DZUS KG14/6/17_176.

38. *Periphyllus californiensis* (Shinji, 1917): London, Kew Garden, UK, 9.6.2017, *Acer palmatum* 'Bi Hoo', ARBN, 3 apt. viv., K. Wieczorek, DZUS KG9/6/17_61; *Acer palmatum,* 2 apt. viv., DZUS KG9/6/17_62.

39. *Periphyllus hirticornis* (Walker, 1848): London, Kew Garden, UK, 14.6.2017, *Acer campestre*, AColl., 2 apt. viv., K. Wieczorek, DZUS KG14/6/17_178.

40. *Periphyllus lyropictus* (Kessler, 1886): London, Kew Garden, UK, 14.6.2017, *Acer campestre*, AColl., 2 apt. viv., K. Wieczorek, DZUS KG14/6/17_180.

41. *Periphyllus testudinaceus* (Fernie, 1852): London, Kew Garden, UK, 9.6.2017, *Acer palmatum* 'Senkaki', ARBN, 3 apt. viv., K. Wieczorek, DZUS KG9/6/17_59; *Acer palmatum* 'Bi Hoo', 3 apt. viv., DZUS KG9/6/17_60; *Acer palmatum*, 2 apt. viv., DZUS KG9/6/17_64; London, Kew Garden, UK, 10.6.2017, *Aesculus* x hybrida, 2 al. viv., K. Wieczorek, DZUS KG10/6/17_75; London, Kew Garden, UK, 14.6.2017, *Acer oliverianum*, AColl., 3 apt. viv., K. Wieczorek, DZUS KG14/6/17_175; *Acer negundo*, AColl., 1 apt. viv., 1 al. viv., DZUS KG14/6/17_177.

APHIDIDAE:APHIDINAE: Aphidini

42. *Aphis* (*Aphis*) *fabae* Scopoli, 1763: London, Kew Garden, UK, 6.6.2017, *Yucca* sp., TRON, 3 apt. viv., K. Wieczorek, DZUS KG6/6/17_3; London, Kew Garden, UK, 9.6.2017, *Celastrus orbiculatus*, ARBN, 3 apt. viv., K. Wieczorek, DZUS KG9/6/17_52; London, Kew Garden, UK, 9.6.2017, *Polyspora* sp., ARBN, 3 apt. viv., K. Wieczorek, DZUS KG9/6/17_63; London, Kew Garden, UK, 9.6.2017, *Koelreuteria bipinnata*, 3 apt. viv., K. Wieczorek, DZUS KG9/6/17_73; London, Kew Garden, UK, 10.6.2017, *Monarda fistulosa* var *menthifolia*, RG, 3 apt. viv., K. Wieczorek, DZUS KG10/6/17_88; *Ageratina ligustrina*, 3 apt. viv., DZUS KG10/6/17_89; London, Kew Garden, UK, 10.6.2017, *Echium amoenum*, RG, 3 apt. viv., K. Wieczorek, DZUS KG10/6/17_94; *Erythranthe naiandina*, 2 apt. viv., DZUS KG10/6/17_95; London, Kew Garden, UK, 11.6.2017, *Rheum palmatum*, PFB, 3 apt. viv., K. Wieczorek, DZUS KG11/6/17_97, *Cistus laurifolius*, 3 apt. viv., DZUS KG11/6/17_101, *Vicia faba*, SVP, 2 apt. viv., 1 al. viv., DZUS KG11/6/17_102, *Solanum tuberosum*, SVP, 3 apt. viv., DZUS KG11/6/17_109; DZUS KG11/6/17_110, *Rheum rhabarbarum* PFB, 2 apt. viv., 1 al. viv., DZUS KG11/6/17_117, *Iris* sp., PFB, 2 apt. viv., DZUS KG11/6/17_118, *Eschscholzia californica*, PFB, 3 apt. viv., DZUS KG11/6/17_120; London, Kew Garden, UK, 12.6.2017, *Viburnum* sp., ARBN, 2 apt. viv., 1 al. viv., DZUS KG12/6/17_125; *Silybum marianum*, PFB, 3 apt. viv., DZUS KG12/6/17_134; *Musa* sp., 1 apt. viv., 1 al. viv., DZUS KG12/6/17_141; 3 apt. viv., DZUS KG12/6/17_142; *Hedera* sp., 1 apt. viv., DZUS KG12/6/17_143; *Cistus laurifolius*, MG, 3 apt. viv., DZUS KG13/6/17_148; London, Kew Garden, UK, 13.6.2017, *Arctium lappa*, L, 2 apt. viv., K. Wieczorek, DZUS KG13/6/17_152; London, Kew Garden, UK, 14.6.2017, *Pieris japonica*, JG, 3 apt. viv., K. Wieczorek, DZUS KG14/6/17_164, *Fatsia japonica*, JG, 2 apt. viv., 1 al. viv., DZUS KG14/6/17_165; *Viburnum farreri*, JG, 2 apt. viv., DZUS KG14/6/17_166; London, Kew Garden, UK, 15.6.2017, *Achillea* sp., the Great Broad Walk Borders, 2 apt. viv., DZUS KG15/6/17_191, *Rudbeckia* sp., 2 apt. viv., DZUS KG15/6/17_192; *Achillea* sp., 1 apt. viv., DZUS KG15/6/17_193, *Aesculus turbinata*, 1 apt. viv., 1 al. viv., DZUS KG15/6/17_196; London, Kew Garden, UK, 16.6.2017, *Yucca glauca*, RG, 2 apt. viv., DZUS KG16/6/17_206, *Rheum palmatum*, near NO, 2 apt. viv., DZUS KG16/6/17_207, *Leucanthemum* x *superbum*, NO, 1 apt. viv., 1 al. viv., DZUS KG16/6/17_209, *Cynara cardunculus*, RK, 2 apt. viv., DZUS KG16/6/17_215, *Digitalis purpurea*, RK, 2 apt. viv., 1 al. viv., DZUS KG16/6/17_216.

43. *Aphis* (*Aphis*) *farinosa* Gmelin, 1790: London, Kew Garden, UK, 12.6.2017, *Salix aegyptiaca*, L, 3 apt. viv., K. Wieczorek, DZUS KG12/6/17_132; London, Kew Garden, UK, 13.6.2017, *Salix prolixa*, L, 2 apt. viv., K. Wieczorek, DZUS KG13/6/17_157.

44. *Aphis* (*Aphis*) *gossypii* Glover, 1877: London, Kew Garden, UK, 6.6.2017, *Oxylobium lineare*, TRON, 2 apt. viv., K. Wieczorek, DZUS KG6/6/17_6; London, Kew Garden, UK, 13.6.2017, *Leptodermis pilosa*, MG, 3 apt. viv., K. Wieczorek, DZUS KG13/6/17_145.

45. *Aphis* (*Aphis*) *ilicis* Kaltenbach, 1843: London, Kew Garden, UK, 16.6.2017, *Ilex* sp., RK, 2 apt. viv., DZUS KG16/6/17_214.

46. *Aphis* (*Aphis*) *newtoni* Theobald, 1927: London, Kew Garden, UK, 16.6.2017, *Iris* sp., NO, 2 apt. viv., 1 al. viv., DZUS KG16/6/17_211.

47. *Aphis* (*Aphis*) *pomi* De Geer, 1773: London, Kew Garden, UK, 9.6.2017, *Crataegus pentagyna*, ARBN, 3 apt. viv., K. Wieczorek, DZUS KG9/6/17_53, *Hedlundia minima*, 1 apt. viv., 2 al. viv., DZUS KG9/6/17_56; London, Kew Garden, UK, 10.6.2017, *Malus tschonoskii*, 3 apt. viv., K. Wieczorek, DZUS KG10/6/17_81.

48. *Aphis* (*Aphis*) *sedi* Kaltenbach, 1843: London, Kew Garden, UK, 11.6.2017, *Sedum telephium*, PFB, 2 apt. viv., K. Wieczorek, DZUS KG11/6/17_98.

49. *Aphis* (*Aphis*) *spiraecola* Patch, 1914: London, Kew Garden, UK, 6.6.2017, *Bremeria landia* var. *holosericea* , TRON, 3 apt. viv., K. Wieczorek, DZUS KG6/6/17_7; London, Kew Garden, UK, 14.6.2017, *Rhododendron* sp., JG, 3 apt. viv., K. Wieczorek, DZUS KG14/6/17_169.

50. *Aphis* (*Aphis*) *verbasci* Schrank, 1801: London, Kew Garden, UK, 11.6.2017, *Verbascum densiflorum*, PFB, 2 apt. viv., 1 al. viv., K. Wieczorek, DZUS KG11/6/17_121.

51. *Aphis* (*Bursaphis*) *grossulariae* Kaltenbach, 1843: London, Kew Garden, UK, 10.6.2017, *Oenothera magellanica*, RG, 1 apt. viv., 1 al. viv., K. Wieczorek, DZUS KG10/6/17_87; London, Kew Garden, UK, 11.6.2017, *Ribes* sp., SVP, 3 apt. viv., K. Wieczorek, DZUS KG11/6/17_103.

52. *Aphis* (*Toxoptera*) *aurantii* Boyer De Fonscolombe, 1841: London, Kew Garden, UK, 10.6.2017, *Camellia japonica*, 1 apt. viv., K. Wieczorek, DZUS KG10/6/17_81.

53. *Hyalopterus pruni* (Geoffroy, 1762): London, Kew Garden, UK, 12.6.2017, Poaceae, AG, 2 apt. viv., 1 al. viv., K. Wieczorek, DZUS KG12/6/17_140; London, Kew Garden, UK, 13.6.2017, *Phragmites australis*, L, 3 apt. viv., K. Wieczorek, DZUS KG13/6/17_151.

54. *Rhopalosiphum nymphaeae* (Linnaeus, 1761): London, Kew Garden, UK, 12.6.2017, *Acorus calamus* 'Variegata', AG, 2 apt. viv., K. Wieczorek, DZUS KG12/6/17_138, *Iris* sp., 2 apt. viv., DZUS KG12/6/17_139.

APHIDIDAE:APHIDINAE: Macrosiphini

55. *Acyrthosiphon* (*Acyrthosiphon*) *malvae* (Mosley, 1841): London, Kew Garden, UK, 10.6.2017, *Clianthus puniceus*, DG, 2 apt. viv., K. Wieczorek, DZUS KG10/6/17_86.

56. *Acyrthosiphon* (*Acyrthosiphon*) *pisum* Harris, 1776: London, Kew Garden, UK, 11.6.2017, *Lathyrus* sp., PFB, 1 apt. viv., 1 al. viv., K. Wieczorek, DZUS KG11/6/17_96.

57. *Brachycaudus* (*Acaudus) lychnidis* (Linnaeus, 1758): London, Kew Garden, UK, 11.6.2017, *Lychnis coronaria*, PFB, 3 apt. viv., K. Wieczorek, DZUS KG11/6/17_116.

58. *Brachycaudus* (*Prunaphis*) *cardui* (Linnaeus, 1758): London, Kew Garden, UK, 10.6.2017, *Matricaria chamomilla*, RG, 2 apt. viv., 1 al. viv., K. Wieczorek, DZUS KG10/6/17_90; London, Kew Garden, UK, 11.6.2017, *Achillea millefolium* 'Pink Grapefruit', PFB, 1 apt. viv., K. Wieczorek, DZUS KG11/6/17_107; London, Kew Garden, UK, 16.6.2017, *Leucantheum* x *superbum*, NO, 2 apt. viv., DZUS KG16/6/17_208.

59. *Cavariella aegopodii* (Scopoli, 1763): London, Kew Garden, UK, 10.6.2017, *Foeniculum vulgare*, DG, 3 apt. viv., K. Wieczorek, DZUS KG10/6/17_83.

60. *Chaetosiphon* (*Pentatrichopus*) *fragaefolii* (Cockerell, 1901): London, Kew Garden, UK, 11.6.2017, *Fragaria* x *ananassa*, PFB, 1 apt. viv., K. Wieczorek, DZUS KG11/6/17_100.

61. *Corylobium avellanae* (Schrank, 1801): London, Kew Garden, UK, 10.6.2017, *Corylus avellana*, 1 apt. viv., K. Wieczorek, DZUS KG10/6/17_78.

62. *Cryptomyzus* (*Cryptomyzus*) *korschelti* Bőrner, 1938: London, Kew Garden, UK, 12.6.2017, *Ribes orientale,* ARBN, 2 apt. viv., K. Wieczorek, DZUS KG12/6/17_124.

63. *Cryptomyzus* (*Cryptomyzus*) *ribis* (Linnaeus, 1758): London, Kew Garden, UK, 16.6.2017, *Ribes nigrum*, RK, 2 apt. viv., K. Wieczorek, DZUS KG16/6/17_217.

64. *Dysaphis* (*Dysaphis*) *tulipae* (Boyer de Fonscolombe, 1841): London, Kew Garden, UK, 12.6.2017, *Iris* sp, AG, 2 apt. viv., K. Wieczorek, DZUS KG12/6/17_137.

65. *Dysaphis* (*Pomaphis*) *plantaginea* (Passerini, 1860): London, Kew Garden, UK, 11.6.2017, *Malus domestica*, SVP, 2 apt. viv., 1 al. viv., K. Wieczorek, DZUS KG11/6/17_104.

66. *Ericolophium holsti* (Takahashi, 1935): London, Kew Garden, UK, 8.6.2017, *Rhododendron* sp., RD, 1 al. viv., K. Wieczorek, DZUS KG8/6/17_39; 1 al. viv. DZUS KG8/6/17_42; 2 al. viv., DZUS KG8/6/17_43.

67. *Hyadaphis passerinii* (Del Guercio, 1911): London, Kew Garden, UK, 10.6.2017, *Lonicera implexa*, DG, 1 apt. viv., 1 al. viv., K. Wieczorek, DZUS KG10/6/17_85.

68. *Illinoia* (*Illinoia*) *liriodendri* (Monell, 1879): London, Kew Garden, UK, 13.6.2017, *Liriodendron tulipifera*, near Palm House, 2 apt. viv., 1 al. viv., K. Wieczorek, DZUS KG13/6/17_150.

69. *Illinoia* (*Illinoia*) *morrisoni* (Swain, 1918): London, Kew Garden, UK, 15.6.2017, *Sequoia sempervirens*, ReG, 2 apt. viv., 1 al. viv., K. Wieczorek, DZUS KG15/6/17_181.

70. *Illinoia* (*Masonaphis*) *lambersi* (Mac Gillivray, 1960): London, Kew Garden, UK, 9.6.2017, *Rhododendron* 'Golden Sunset', RD, 3 al. viv., K. Wieczorek, DZUS KG9/6/17_72.

71. *Macrosiphoniella* (*Macrosiphoniella*) *absinthii* (Linnaeus, 1758): London, Kew Garden, UK, 11.6.2017, *Achillea millefolium* 'Pink Grapefruit', PFB, 2 al. viv., K. Wieczorek, DZUS KG11/6/17_119; London, Kew Garden, UK, 13.6.2017, *Artemisia absinthium*, MG, 3 apt. viv., K. Wieczorek, DZUS KG13/6/17_147.

72. *Macrosiphoniella* (*Macrosiphoniella*) *millefolii* (De Geer, 1773): London, Kew Garden, UK, 13.6.2017, *Achillea* sp., MG, 2 al. viv., K. Wieczorek, DZUS KG13/6/17_146.

73. *Macrosiphum* (*Macrosiphum*) *albifrons* Essig, 1911: London, Kew Garden, UK, 11.6.2017, *Lupinus* 'My Castle', PFB, 1 al. viv., K. Wieczorek, DZUS KG11/6/17_99a, 2 apt. viv.. DZUS KG11/6/17_99b, *Lupinus ehrenbergii*, 2 apt. viv., DZUS KG11/6/17_114, *Lupinus* 'The Governor', 2 apt. viv., 1 al. viv., DZUS KG11/6/17_115.

74. *Macrosiphum* (*Macrosiphum*) *euphorbiae* (Thomas, 1878): London, Kew Garden, UK, 6.6.2017, *Nelumbo nucifera*, TRON, 3 apt. viv., K. Wieczorek, DZUS KG6/6/17_2; London, Kew Garden, UK, 6.6.2017, *Yucca* sp., TRON, 2 apt. viv., 1 al. viv., K. Wieczorek, DZUS KG6/6/17_4; London, Kew Garden, UK, 6.6.2017, *Wahlenbergia angustifolia*, TRON, 3 apt. viv., K. Wieczorek, DZUS KG6/6/17_5; London, Kew Garden, UK, 9.6.2017, *Cornus mas*, ARBN, 2 apt. viv., K. Wieczorek, DZUS KG9/6/17_65; *Skimia* sp., 3 apt. viv., DZUS KG9/6/17_66; London, Kew Garden, UK, 11.6.2017, *Solanum lycopersicum*, SVP, 3 apt. viv., K. Wieczorek, DZUS KG11/6/17_108; *Iris* sp., PFB, 3 apt. viv., DZUS KG11/6/17_113; London, Kew Garden, UK, 12.6.2017, *Iris* sp., AG, 2 apt. viv., K. Wieczorek, DZUS KG12/6/17_136; London, Kew Garden, UK, 16.6.2017, *Iris pallida*, NO, 1 apt. viv., 1 al. viv., K. Wieczorek, DZUS KG16/6/17_210, *Paulownia fargesii*, NO, 2 apt. viv., DZUS KG16/6/17_211, *Aquilegia vulgaris*, RK, 2 apt. viv., DZUS KG16/6/17_213.

75. *Macrosiphum* (*Macrosiphum*) *euphorbiellum* Theobald, 1917: London, Kew Garden, UK, 10.6.2017, *Euphorbia characias*, DG, 2 apt. viv., K. Wieczorek, DZUS KG10/6/17_84.

76. *Macrosiphum* (*Macrosiphum*) *hellebori* Theobald & Walton, 1923: London, Kew Garden, UK, 10.6.2017, *Sedum telephium*, RG, 1 al. viv., K. Wieczorek, DZUS KG10/6/17_92.

77. *Macrosiphum* (*Macrosiphum*) *rosae* (Linnaeus, 1758): London, Kew Garden, UK, 12.6.2017, *Rosa* sp., PFB, 1 apt. viv., K. Wieczorek, DZUS KG12/6/17_135; London, Kew Garden, UK, 15.6.2017, *Rosa* 'Jacques Cartier', RoG, 2 apt. viv., K. Wieczorek, DZUS KG15/6/17_200, *Rosa* 'Tuscany', RoG, 1 apt. viv., DZUS KG15/6/17_201, *Rosa* sp., RoG, 2 apt. viv., DZUS KG15/6/17_202, *Rosa* sp., RoG, 2 apt. viv., DZUS KG15/6/17_203, *Rosa* sp., RoG, 2 apt. viv., DZUS KG15/6/17_204.

78. *Megoura viciae* Buckton, 1876: London, Kew Garden, UK, 11.6.2017, *Lathyrus montanus*, SVP, 2 apt. viv., K. Wieczorek, DZUS KG11/6/17_111.

79. *Melanaphis pyraria* (Passerini, 1861): London, Kew Garden, UK, 11.6.2017, *Pyrus* sp., SVP, 3 apt. viv., K. Wieczorek, DZUS KG11/6/17_106.

80. *Myzus* (*Myzus*) *cerasi* (Fabricius, 1775): London, Kew Garden, UK, 9.6.2017, *Prunus serrulata* 'Amanagawa', ARBN, 1 apt. viv., 1 al. viv., K. Wieczorek, DZUS KG9/6/17_57.

81. *Myzus* (*Myzus*) *ornatus* Laing, 1932: London, Kew Garden, UK, 9.6.2017, *Primula* sp., ARBN, 3 apt. viv., K. Wieczorek, DZUS KG9/6/17_49; *Primula* sec. Proliferae, 3 apt. viv., DZUS KG9/6/17_50; *Saurauia napaulensis*, 2 apt. viv., 1 al. viv., DZUS KG9/6/17_51.

82. *Myzus* (*Nectarosiphon*) *persicae* (Sulzer, 1776): London, Kew Garden, UK, 6.6.2017, *Crossandra pungens*, TRON, 2 apt. viv., K. Wieczorek, DZUS KG6/6/17_1; London, Kew Garden, UK, 9.6.2017, *Prunus* x *yedoensis*, ARBN, 3 apt. viv., K. Wieczorek, DZUS KG9/6/17_58.

83. *Neotoxoptera formosana* (Takahashi, 1921): London, Kew Garden, UK, 10.6.2017, *Allium nutans*, RG, 3 apt. viv., K. Wieczorek, DZUS KG10/6/17_91.

84. *Pterocomma pilosum* Buckton, 1879: London, Kew Garden, UK, 13.6.2017, *Salix lasiolepis*, L, 2 apt. viv., K. Wieczorek, DZUS KG13/6/17_158.

85. *Pterocomma populeum* (Kaltenbach, 1843): London, Kew Garden, UK, 7.6.2017, *Populus nigra betulifolia*, PColl., 3 apt. viv., K. Wieczorek, DZUS KG7/6/17_21, 9.6.2017, ARBN, 3 apt. viv., K. Wieczorek, DZUS KG9/6/17_67; London, Kew Garden, UK, 7.6.2017, *Populus incrassata*, PColl., 3 apt. viv., K. Wieczorek, DZUS KG7/6/17_23; London, Kew Garden, UK, 7.6.2017, *Populus grandidentata*, PColl., 2 apt. viv., 1 al. viv., K. Wieczorek, DZUS KG7/6/17_25; London, Kew Garden, UK, 8.6.2017, *Populus* x *canadensis*, PColl., 1 al. viv., K. Wieczorek, DZUS KG8/6/17_48; London, Kew Garden, UK, 9.6.2017; London, Kew Garden, UK, 15.6.2017, *Populus balsamifera*, PColl., 2 apt. viv., K. Wieczorek, DZUS KG15/6/17_188, *Populus nigra*, PColl., 3 apt. viv., DZUS KG15/6/17_198.

LACHNINAE: Eulachnini

86. *Cinara* (*Cinara*) *cedri* Mimeur, 1936: London, Kew Garden, UK, 8.6.2017, *Cedrus libani*, 3 apt. viv., K. Wieczorek, DZUS KG8/6/17_32; London, Kew Garden, UK, 14.6.2017, *Cedrus atlantica*, near Palm House, 1 apt. viv., K. Wieczorek, DZUS KG14/6/17_162, *Cedrus libani*, JG, 2 apt. viv., K. Wieczorek, DZUS KG14/6/17_163.

87. *Cinara* (*Cinara*) *piceae* (Panzer, 1800): London, Kew Garden, UK, 14.6.2017, *Picea* sp., Pi, 1 al. viv., K. Wieczorek, DZUS KG14/6/17_170.

88. *Cinara* (*Cinara*) *pilosa* (Zetterstedt, 1940): London, Kew Garden, UK, 10.6.2017, *Pinus sylvestris* 'Beuvronensis', RG, 2 al. viv., K. Wieczorek, DZUS KG10/6/17_93.

89. *Cinara* (*Cinara*) *pinea* (Mordvilko, 1895): London, Kew Garden, UK, 14.6.2017, *Pinus sylvestris*, JG, 3 apt. viv., K. Wieczorek, DZUS KG14/6/17_168.

90. *Cinara* (*Cinara*) *pini* (Linnaeus, 1758): London, Kew Garden, UK, 15.6.2017, *Pinus nigra*, Pi., 1 apt. viv., 1 al. viv., K. Wieczorek, DZUS KG15/6/17_182, *Pinus patula*, Pi., 1 apt. viv., DZUS KG15/6/17_183.

91. *Cinara* (*Schizolachnus*) *pineti* (Fabricius, 1781): London, Kew Garden, UK, 14.6.2017, *Pinus nigra*, JG, 1 apt. viv., K. Wieczorek, DZUS KG14/6/17_167.

LACHNINAE: Lachnini

92. *Lachnus pallipes* (Hartig, 1841): London, Kew Garden, UK, 8.6.2017, *Quercus cornelius-mulleri*, QColl., 2 al. viv., K. Wieczorek, DZUS KG8/6/17_35, 1 apt. viv. DZUS KG8/6/17_36; London, Kew Garden, UK, 10.6.2017, *Quercus palustris*, 1 al. viv., K. Wieczorek, DZUS KG10/6/17_76; 1 apt. viv DZUS KG10/6/17_77.

93. *Lachnus roboris* (Linnaeus, 1758): London, Kew Garden, UK, 7.6.2017, *Quercus falcata*, QColl., 2 apt. viv., K. Wieczorek, DZUS KG7/6/17_29; London, Kew Garden, UK, 8.6.2017, *Quercus pontica*, QColl., 1 apt. viv., K. Wieczorek, DZUS KG8/6/17_37, 1 al. viv., DZUS KG8/6/17_38; London, Kew Garden, UK, 9.6.2017, *Quercus* sp., ARBN, 1 al. viv., K. Wieczorek, DZUS KG9/6/17_54; London, Kew Garden, UK, 15.6.2017, *Quercus faginea*, near Play Ground, 1 apt. viv., 1 al. viv., K. Wieczorek, DZUS KG15/6/17_184, *Quercus* x *sargentii* 'Thomas', 1 apt. viv., DZUS KG15/6/17_195; London, Kew Garden, UK, 16.6.2017, *Quercus nigra*, 2 apt. viv., K. Wieczorek, DZUS KG16/6/17_219.

94. *Maculolachnus submacula* (Walker, 1848): London, Kew Garden, UK, 11.6.2017, *Rosa* sp., PFB, 1 apt. viv., K. Wieczorek, DZUS KG11/6/17_112; London, Kew Garden, UK, 15.6.2017, *Rosa* sp., RoG, 2 apt. viv., K. Wieczorek, DZUS KG15/6/17_199.
